# Nanomedicine and the innate immune system: immunogenicity, nanoparticle-immune cell interactions and implications for cancer immunotherapy

**DOI:** 10.3389/fimmu.2026.1946595

**Published:** 2026-10-20

**Authors:** Giuseppina Bozzuto, Annarita Stringaro, Sofia Migani, Marisa Colone, Annarica Calcabrini, Maria Luisa Dupuis

**Affiliations:** 1 National Center for Drug Research and Evaluation, Italian National Institute of Health, Rome, Italy; 2 School of Science and Technology, Chemistry Division, University of Camerino, Camerino, Italy

**Keywords:** immunogenicity, innate immunity, nanomedicine, nanoparticles, tumor microenvironment

## Abstract

Nanoparticles were initially considered inert carriers for therapeutic payloads. This view has evolved substantially, as a growing body of experimental and clinical evidence indicates that engineered nanoparticles act as active immunomodulators, engaging pattern recognition receptors (PRRs), activating the complement cascade, reprogramming macrophage polarization, and inducing trained immunity in innate immune cells. This dual role of nanoparticles as both immunological agents and delivery vehicles represents a fundamental change that demands an integrative framework combining immunogenicity assessment, nanotoxicology, and therapeutic exploitation. This review provides an overview of the current state of the art at the nanomedicine–innate immunity interface across six nanoparticle classes, adopting a comparative framework that identifies both the immunological mechanisms shared among platforms and the specific features that distinguish individual nanoparticle systems. The analysis focuses on key innate immune populations, including dendritic cells (DCs), macrophages, natural killer (NK) cells, neutrophils and mast cells, as well as major innate immune signaling pathways, including the cyclic guanosine monophosphate-adenosine monophosphate synthase-stimulator of interferon genes (cGAS–STING) pathway, Toll-like receptor (TLR) signaling, and the NOD-like receptor protein 3 (NLRP3) inflammasome. The innate immune system represents a primary determinant of the biological outcome of nanomaterial exposure and provides a critical link to subsequent adaptive responses. Two methodological themes recur throughout the analysis. The first concerns attribution: distinguishing responses driven by the carrier itself from those driven by its cargo or by contaminants requires defined controls, and in their absence an observed response should not be ascribed to the nanomaterial. The second is inter-individual variability, which emerges as a determinant of outcome rather than as experimental noise, and which appears to arise from the combination of recipient and platform rather than from either alone, so that susceptibility established for one platform does not transfer to another. Our aim is to highlight unexplored connections and experimental gaps that currently limit clinical translation, offering an integrated interpretation of nanoparticle–innate immunity interactions that points to unresolved immunological challenges and oncology-relevant therapeutic opportunities for each class still awaiting validation.

## 1 Introduction

Nanomedicine, defined as the application of nanotechnology to disease prevention, diagnosis and therapy, has matured substantially over the past two decades, evolving from an emerging discipline into an established component of translational biomedical research. Nanoparticulate systems ranging from 1 to 1000 nm, built from organic materials (lipids, polymers, proteins), inorganic compounds (silica, metal oxides), metals (gold, silver, copper) and biological structures (exosomes, extracellular vesicles), are now employed across a broad spectrum of clinical and preclinical applications (1). The clinical approval of formulations such as liposomal doxorubicin (Doxil®/Caelyx®) (2), albumin-bound paclitaxel (Abraxane®), and, more recently, the mRNA lipid nanoparticle vaccines against SARS-CoV-2 (Comirnaty® and Spikevax®) (3) has confirmed the translational potential of nanomedicine (4). These approvals, however, capture only a fraction of ongoing activity: between 50 and 80 formulations have been approved by regulatory agencies worldwide (5), while a systematic analysis of ClinicalTrials.gov has identified over 4,000 trials involving nanomedicines, a 38% increase in recent years (6). Oncology remains the dominant field, accounting for 30% of these trials, but applications in infectious disease, driven by the rise of mRNA vaccines, are expanding rapidly (6).

One aspect of this progress that continues to attract sustained interest is how nanoparticles interact with the host immune system, particularly the innate immune system. Innate immunity provides the first line of host defense through a coordinated network of cells and soluble mediators, including macrophages, dendritic cells (DCs), natural killer (NK) cells, neutrophils, eosinophils, basophils, mast cells, and the complement system, as well as pattern recognition receptors (PRRs) such as Toll-like receptors (TLRs), NOD-like receptors (NLRs), RIG-I-like receptors (RLRs), C-type lectin receptors (CLRs), and cytosolic DNA-sensing pathways including the cyclic guanosine monophosphate-adenosine monophosphate synthase-stimulator of interferon genes (cGAS-STING) axis (7–10). Far from behaving as inert counterparts on contact with nanomaterials, these elements actively recognize, internalize, process and respond to administered nanoparticles, shaping both efficacy and safety (7, 11).

For many years, the immune response to nanoparticles was considered mainly a safety problem, with adverse effects to be mitigated through surface engineering strategies such as PEGylation (polyethylene glycol coating) (11, 12). That framing has been substantially revised: over the past decade, nanoparticles have been reinterpreted not as inert vehicles but as active modulators of immune signaling, capable of amplifying or silencing selected pathways depending on composition, surface architecture, and route of administration (13–16). The shift from “drug carrier” to “immune amplifier” has been documented across multiple nanoparticle classes and has opened new avenues for vaccine design, adjuvant development and a broad range of oncology applications (17, 18).

A further layer of complexity emerged with the recognition that the innate immune system carries a form of memory, trained immunity, sustained by epigenetic and metabolic reprogramming of monocytes and macrophages following prior stimulation (19–21). The ability of nanoparticles to elicit such reprogramming adds a layer of complexity: their immunological imprint may persist long after the particles themselves have been cleared (19, 20, 22).

In parallel, the landscape of cancer immunotherapy has been markedly reshaped by immune checkpoint inhibitors (anti-PD-1, anti-PD-L1, anti-CTLA-4) and CAR-T cell therapies. Most patients, however, do not respond to these treatments, largely because the tumor microenvironment (TME) is structurally immunosuppressive, with M2-polarized tumor-associated macrophages (TAMs) dominating the infiltrate, widespread T-cell exhaustion, and hypoxia, acidosis, and a dense extracellular matrix that further restricts effector function (23–26). Nanoparticles that act on innate immunity within the TME, repolarizing TAMs, licensing DCs, enhancing NK cytotoxicity, and engaging the cGAS–STING axis, represent a complementary strategy for reshaping these suppressive immune landscapes (17, 27–31). This review takes a comparative approach across six nanoparticle classes: liposomes and lipid nanoparticles (LNP); exosomes and plant-derived vesicle-like nanoparticles (PVLN); polymeric nanocapsules; gold nanoparticles and nanorods; copper-based nanoparticles and nanoplatforms; and phytochemical-loaded nanoformulations. For each class, four aspects are examined: (i) physicochemical properties relevant to immune interaction; (ii) mechanisms of innate recognition and activation; (iii) the desired and undesired immunogenicity profile; and (iv) emerging oncology applications, with a particular focus on remodeling of the immunosuppressive tumor microenvironment. Rather than reproducing the detailed coverage already provided in single-class reviews, the goal is to extract mechanistic principles that recur across classes, identify class-specific differences, and provide an integrated interpretation that highlights translational opportunities and unresolved knowledge gaps in the field.

The literature search strategy, including the database and period searched, the search terms used for each nanoparticle class and innate immune pathway, and the inclusion and exclusion criteria applied, is reported in full in the **Supplementary Material**. This is a narrative and comparative review rather than a systematic review: therefore, no formal risk-of-bias assessment, quantitative synthesis, or meta-analysis was undertaken, and a PRISMA flow diagram was not considered applicable.

## 2 Physicochemical properties of nanoparticles and their immunological relevance

The way a nanoparticle engages the innate immune system is strongly influenced by its physicochemical properties, including size, shape, surface charge, chemical composition, mechanical rigidity, hydrophobicity, and surface functionalization (7, 8, 11, 32). Although these properties are often discussed individually, they act in concert. For example, changes in size or shape alter protein corona deposition, receptor recognition, and cellular uptake, while surface chemistry modulates interactions with immune cells and biomolecules. Thus, the immunological behavior of a real nanoparticle is the result of the combined effects of multiple physicochemical properties (**Table 1**).

**Table 1:** Physicochemical properties of nanoparticles and their impact on the innate immune system.

Property	Typical range	Effect on innate immunity	Mechanism	Ref.
Size	1–1000 nm	Modulate uptake, lymphatic drainage, and cytokine response	Size-dependent endocytosis; renal clearance (<5.5 nm) or hepatic (>200 nm)	(7, 11)
Shape	Spherical, discoidal, rod, star	Influences the internalization rate and M1/M2 polarization	Membrane curvature, cellular metabolism	(32, 40)
Charge (ζ)	−50 to +50 mV	Cationic: high uptake, more toxic; anionic: low uptake, less activation	Electrostatic interaction with membrane; corona composition	(13, 16)
Composition	Lipids, polymers, metals, biologicals	Determines biodegradability, specific PRR interaction	TLR recognition, STING activation, cuproptosis	(44, 45)
PEGylation	2–5 kDa PEG	Reduces opsonization; anti-PEG and CARPA risk	Steric shielding; anti-PEG antibodies	(50, 52)
Rigidity	KPa–GPa	Rigid: more uptake; soft: phagocytic evasion	Mechanosensitive ion channels; membrane deformation	(40)
Biomolecular corona	Hard + soft corona	Determines biological identity; opsonization; targeting	Protein adsorption; complement (C3, C1q)	(56, 57)

Properties are discussed individually for clarity, but act in combination; the immunological outcome reflects their joint effect rather than any single parameter.

### 2.1 Size

Size is one of the most influential physicochemical parameters, although the ranges over which it operates are narrower and more model-specific than is often stated. In mice given subcutaneous PLGA–PEG particles, lymphatic trafficking was size-dependent within the sub-100-nanometer range: 20 nm constructs drained rapidly across proximal and distal nodes and were retained there, 40 nm constructs less efficiently, and 100 nm constructs negligibly (7, 11, 33). Renal filtration requires a hydrodynamic diameter below approximately 5.5 nm, a threshold defined in rats using quantum dots with zwitterionic or neutral coatings; with other coatings, serum protein adsorption increased hydrodynamic diameter by more than 15 nm and abolished urinary excretion, indicating that the relevant parameter is the hydrodynamic size, including changes caused by adsorbed proteins (11, 34).

Larger particles tend to be sequestered by the mononuclear phagocyte system, particularly in liver and spleen, although the extent varies with composition, surface properties, shape and host immune phenotype. Mouse strains polarized toward Th1 clear nanoparticles more slowly than Th2-prone strains, an effect mediated by granulocytes and macrophages: macrophages from Th1 strains take up fewer particles in vitro, M2 differentiation increases uptake and M1 reduces it. The same relationship holds in human monocyte-derived macrophages, indicating that clearance depends as much on the immune status of the recipient as on the particle (35).

Size also shapes the quality of the immune response, although the relationship is neither defined by sharp thresholds nor readily transferable between systems. Particles above approximately 100 nm have been reported to favor type 2 responses and particles around 50 nm type 1 responses (36), a generalization drawn from several experimental systems. In mice, ovalbumin-conjugated polystyrene beads gave the strongest IFN-γ responses at 40–49 nm and higher IL-4 responses at 93–123 nm (37). Where shape was varied as well, 193 nm spheres elicited a Th1-biased and 1530 nm rods a Th2-biased response, showing that morphology can modify or override the size-associated tendency (38). With some materials the response is not size-polarized at all: chitosan nanoparticles of 83.66 nm mean diameter, a size at which a type 2 shift might be expected, elicited a balanced Th1/Th2 profile, which the authors attributed to the intrinsic immunostimulatory properties of chitosan, including engagement of TLR4 and mannose receptors, rather than to dimensions alone (39).

Macrophage uptake and processing are likewise influenced by size together with morphology, surface chemistry and rigidity, with size-dependent polarization associated with lysosomal stress and with the distinct effects of different diameters on the plasma membrane (7, 11, 32). Size is therefore best regarded as one component of a broader framework that includes composition, surface chemistry, morphology, rigidity, dose, route and host immune phenotype, and its effects must be established for each carrier system rather than inferred from thresholds.

### 2.2 Shape and geometry

Shape modulates these interactions in often counterintuitive ways. Spherical particles are generally internalized by macrophages more rapidly than discoidal, ellipsoidal, or rod-shaped particles. Consistent with this observation, Key and colleagues showed that soft discoidal polymeric nanoconstructs resist macrophage uptake and enhance vascular targeting in tumor (40). Geometry also imparts functional properties unrelated to uptake. Gold nanorods, owing to their high aspect ratio, exhibit plasmonic properties that differ markedly from those of nanospheres and display distinct interactions with biological membranes (32, 41). Shape also feeds back onto the cell, as membrane curvature generated during internalization influences cellular metabolism and, downstream, macrophage polarization (32).

### 2.3 Surface charge

Surface charge, read out as zeta potential, governs the electrostatic interplay between particle and cell membrane and, through it, the composition of the biomolecular corona that ultimately defines the particle’s biological identity. Cationic surfaces are taken up more avidly than anionic or neutral ones, although this enhanced uptake is often accompanied by increased cytotoxicity and complement activation. The ionizable lipids at the core of modern LNPs help to resolve this trade-off: materials such as ALC-0315 and SM-102 remain neutral at physiological pH and become cationic only within the acidic endosome, so that the particle stays inconspicuous in circulation yet escapes the endosome once internalized (13–15). This pH-switching design underlies both the delivery efficiency and the immunological profile of these systems (16).

### 2.4 Chemical composition and surface functionalization

Material chemistry strongly influences biodegradability, biocompatibility, and the range of immune interactions a nanoparticle can engage. PLGA (Poly(lactic-co-glycolic acid)) and PVA (Polyvinyl Alcohol) are widely used because of their biodegradability and well-established biocompatibility (42, 43). Gold nanoparticles are relatively inert in biological environments, but their plasmonic properties enable photothermal and sensing applications that are not achievable with organic carriers (41, 44). Copper-based systems, in contrast, are of interest precisely because they are not inert: they can trigger cuproptosis, a regulated cell-death pathway linked to intracellular copper accumulation and metabolic stress (45–48). Surface coatings add another layer of control, although not always in a straightforward way. PEGylation reduces opsonization and prolongs circulation but can also induce anti-PEG antibodies and complement activation-related pseudoallergy (CARPA) (49–52). The resulting immune-mediated effects on nanoparticle clearance are mechanistically linked to changes in the protein corona: anti-PEG IgM alters the composition of the corona deposited on PEGylated nanoparticles and activates the complement cascade, and this alteration in corona composition, rather than antibody binding itself, accounts for the shortened circulation observed upon re-administration (53). Targeting ligands such as antibodies, peptides, and aptamers can improve specificity, but such modifications can substantially reshape the particle’s immunological profile (54).

### 2.5 Rigidity and mechanical properties

Stiffness is the least intuitive of these parameters and was long overlooked, yet it is now increasingly recognized as one of the most functionally relevant. Both mechanosensitive ion channels and the dynamics of membrane deformation respond to a particle’s elastic modulus, with important functional consequences: rigid particles are generally internalized more efficiently by macrophages, whereas softer ones evade phagocytic surveillance and circulate for longer periods (32, 40). This property can therefore be exploited as a design parameter, particularly in polymeric nanostructures, where formulation strategies and material composition enable the tuning of mechanical properties while simultaneously influencing drug release behavior and immunological responses (40, 55).

## 3 Impact of nanoparticles on the innate immune system

This section provides a mechanistic core of the review, describing how the innate immune system recognizes, processes, and responds to nanoparticles, regardless of class (**Figure 1**). A mechanistic understanding at this level is a prerequisite for the rational design of nanomedicines with predictable immunological profiles and for anticipating the immunological behavior of new formulations.

**Figure 1:**
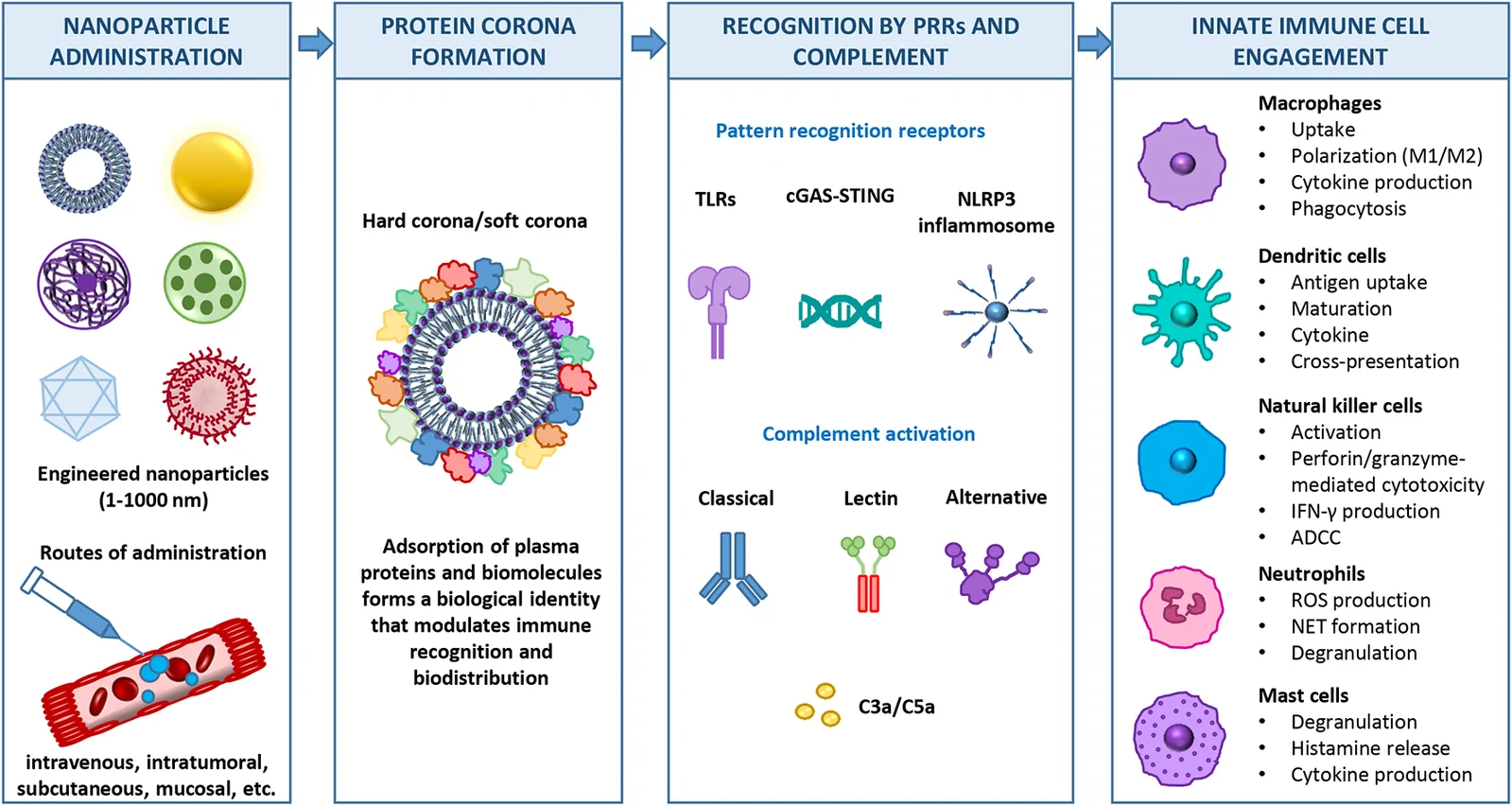
Schematic overview of nanoparticle interactions with the innate immune system. Engineered nanoparticles (1–1000 nm), administered through intravenous, intratumoral, subcutaneous, or mucosal routes, are rapidly coated by plasma proteins and biomolecules, forming a biomolecular corona composed of a stable hard corona and a dynamic soft corona. This corona confers the biological identity that governs immune recognition and biodistribution. The coated nanoparticles are then sensed by pattern recognition receptors (Toll-like receptors, the cytosolic cGAS–STING axis, and the NLRP3 inflammasome) and by the complement system, which can be activated through the classical, lectin, or alternative pathway, generating the anaphylatoxins C3a and C5a. These recognition events engage the principal innate immune cell populations — macrophages (uptake, M1/M2 polarization, cytokine production, phagocytosis), dendritic cells (antigen uptake, maturation, cytokine, cross-presentation), natural killer cells (activation, perforin/granzyme-mediated cytotoxicity, IFN-γ production, ADCC), neutrophils (ROS production, NET formation, degranulation), and mast cells (degranulation, histamine release, cytokine production) — which collectively determine the biological response to nanomedicines.

### 3.1 Biomolecular corona and first contact

On entering a biological environment, nanoparticles are immediately coated with serum proteins, forming the biomolecular corona that largely defines their biological identity and mediates their interactions with the immune system (7, 56, 57). Corona composition depends on both the physicochemical properties of the nanoparticle and the biological microenvironment in which it is deposited. Consequently, it varies substantially between healthy and diseased states, between vascular compartments, and even between individual patients, contributing to inter-individual variability in clinical responses. Liu et al. characterized the nanoparticle–protein corona complex systematically, distinguishing a hard corona (stable, high-affinity proteins directly associated with the surface), a soft corona (dynamic and exchangeable proteins), and an interfacial corona located at the nano–bio interface (56). These compartments are not static. Through the Vroman effect, initially abundant low-affinity proteins such as albumin are progressively displaced by less abundant but higher-affinity species, including immunoglobulins, complement factors, and apolipoproteins, thereby reshaping the immunological signature of the particle over time. Wang and Zhang described the Janus character of the protein corona: it can either mask targeting ligands and reduce nanovector efficacy or favor interactions with immune receptors, raising immunogenicity (57).

Complement proteins (C3, C1q, factor B, properdin) are among the earliest and most consequential corona components. Their deposition can proceed through the classical pathway (C1q recognition of adsorbed immunoglobulins), the lectin pathway (mannose-binding lectin recognition of surface carbohydrates), or the alternative pathway (spontaneous C3 hydrolysis amplified on reactive surfaces); the relative contribution of each pathway depends on surface chemistry. Complement opsonization accelerates clearance by the mononuclear phagocyte system (liver and spleen) and can trigger CARPA, an acute hypersensitivity reaction that has complicated the clinical translation of several nanomedicines, including liposomal formulations (50, 51). Boraschi and collaborators systematically reviewed how nanoparticle properties such as size, shape, and deformability influence cellular uptake and innate immune responses (7). He et al. reports that, depending on nanoparticle design and experimental conditions, the liver may sequester the vast majority of systemically administered nanoparticles, in some cases approaching 99% of the injected dose (58). Consequently, strategies aimed at reducing hepatic sequestration (including optimization of size, morphology, stealth coatings, and active targeting approaches) remain central to improving nanoparticle accumulation at target tissues.

### 3.2 Recognition through innate immunity receptors (PRR)

The innate immune system recognizes nanoparticles through pattern-recognition receptors (PRRs), which evolved to detect pathogen-associated molecular patterns (PAMPs) and damage-associated molecular patterns (DAMPs). Nanoparticles occupy an intermediate conceptual space: although they are neither pathogens nor endogenous damage signals, their surface chemistry, size, and aggregation behavior can generate PAMP-like or DAMP-like signals, thereby promoting PRR engagement through molecular resemblance to structures associated with infection or cellular stress (8, 48). Among the PRRs most prominently implicated in nanoparticle sensing are Toll-like receptors (TLRs), the cGAS–STING pathway, and the NLRP3 inflammasome (8).

#### 3.2.1 Toll-like receptors

Surface TLRs (TLR2, TLR4, TLR6) and endosomal TLRs (TLR3, TLR7, TLR8, TLR9) are among the PRRs most frequently activated by nanoparticles (8). Ionizable lipids in LNPs have been reported to activate TLR4 and other innate immune pathways, promoting downstream dendritic cell maturation and the production of pro-inflammatory cytokines, including TNF-α, IL-6, and IL-1β (13, 15, 16). TLR7 and TLR8 recognize endosomal single-stranded mRNA, including exogenous mRNA, particularly when unmodified, whereas unmethylated CpG-rich DNA can activate TLR9 (16, 59). Downstream, TLR engagement bifurcates into two major signaling branches: the MyD88-dependent pathway, common to most TLRs and responsible for rapid NF-κB-driven transcription of pro-inflammatory cytokines; and the TRIF-dependent pathway, engaged preferentially by TLR3 and TLR4, which drives type I interferon responses via IRF3. The balance between these pathways are an important determinant of whether a nanoparticle elicits a predominantly inflammatory or interferogenic signature (15, 16). Nucleoside modifications such as pseudouridine and N1-methylpseudouridine reduce TLR7/8 activation but do not eliminate innate immunostimulation, which is partly compensated by the intrinsic adjuvant activity of the LNP carrier (13, 15, 16, 59). Silva et al. further demonstrated that the physicochemical properties of nanoparticles influence which PRR pathways are preferentially activated (8).

#### 3.2.2 cGAS–STING pathway

The cGAS–STING pathway (cyclic GMP–AMP synthase–Stimulator of Interferon Genes) has emerged as one of the most intensively studied pathways in nanoparticle-mediated cancer immunotherapy (27, 30). The enzyme cGAS detects cytosolic DNA, both exogenous and endogenous, including mitochondrial DNA released during cellular stress or cell death, and synthesizes the second messenger 2′3′-cGAMP, which subsequently engages STING on the endoplasmic reticulum membrane. STING activation drives phosphorylation of TBK1 and IRF3, culminating in the production of type I interferons (IFN-α, IFN-β) and pro-inflammatory cytokines (TNF-α, IL-6, IL-1β) (27, 60). The resulting microenvironment is strongly immunostimulatory and supports dendritic cell (DC) maturation, NK-cell activation, and T-cell priming (27, 30, 60). The therapeutic appeal of this pathway stems from its ability to simultaneously recruit effector immune cells, restore type I IFN signaling, and promote adaptive antitumor immunity. Historically, however, the major obstacle has been pharmacological, as free STING agonists are rapidly degraded, poorly cell-permeable, and systemically toxic at therapeutically effective doses. Ying et al. provided an in-depth analysis of cGAS–STING signaling and its capacity to modulate the antitumor immune cycle, highlighting how nanomedicines can overcome the biodistribution and toxicity limitations of conventional agonists (27). Metallic nanoparticles containing Mn²^+^ ions (e.g., gold nanorods with MnO_2_ shells) or Cu²^+^ derived from copper-based nanoparticles can enhance cGAS–STING signaling, thereby adding an intrinsic immunostimulatory function to their delivery properties (17, 44). In particular, Mn²^+^ acts as an allosteric sensitizer of cGAS toward cytosolic DNA, lowering the activation threshold by approximately two orders of magnitude and converting subthreshold DNA leakage into productive signaling.

#### 3.2.3 NLRP3 inflammasome

The NLRP3 inflammasome is a cytosolic multiprotein complex that, once assembled, activates caspase-1, leading to the maturation and secretion of IL-1β and IL-18 and, in some contexts, triggering pyroptosis, a lytic and highly inflammatory form of cell death. NLRP3 activation follows a two-signal model: a priming signal (signal-1), typically mediated by TLRs, which upregulates NLRP3 and pro-IL-1β transcription; and an activation signal (signal-2), which triggers inflammasome assembly proper (10). Silica nanoparticles, metal oxides, carbon nanotubes, and several polymeric nanoparticles can serve as signal 2 through multiple convergent mechanisms, including lysosomal rupture following phagocytosis (with consequent cathepsin B release into the cytosol), reactive oxygen species (ROS) generation, potassium efflux, and plasma membrane perturbation (8, 10). Inflammasome activation is intrinsically double-edged: in vaccine settings, it can amplify immunogenicity and promote protective immune responses, whereas under chronic exposure conditions, it may drive persistent inflammation and fibrosis (10, 61). The distinction between these outcomes depends largely on the magnitude, duration, and resolution of activation: transient inflammasome signaling is generally immunogenic, whereas its sustained activation becomes pathogenic.

#### 3.2.4 Determinants of transient versus persistent activation

The conditions that favor transient, adjuvant-scale inflammasome signaling over persistent, pathogenic activation depend on several interrelated factors, including biopersistence, dosing regimen, and material properties. These factors are better understood as qualitative determinants rather than as defined thresholds. Biopersistence and clearance are particularly relevant, as the duration of inflammasome activation is influenced by the persistence of the adjuvant or particulate material at the site of administration. Clinical and experimental vaccine adjuvants, including aluminum salts, saponins, and emulsions, can engage inflammasome-related pathways and promote IL-1 family signaling, thereby contributing to the induction and shaping of adaptive immune responses (62). The role of the inflammasome in responses to nanoparticle adjuvants specifically has been reviewed separately (63). Particles and crystals that resist dissolution and clearance can prolong the cellular stimulus and, when persistent, contribute to chronic inflammatory responses and particle-associated disease (64). The distinction therefore rests less on the administered dose alone than on the residence time of the material in the lysosomal compartment.

The second axis is the dosing regimen. A single exposure that is cleared before the next produces discrete episodes of activation separated by resolution, whereas repeated administration at intervals shorter than the clearance time produces a cumulative burden even when each individual dose is well tolerated. Thus, the biological effect may depend not only on the amount administered at a single time point, but also on the duration and temporal pattern of the resulting cellular burden.

The third axis comprises the material properties that govern the intensity of the signal-2 event itself, which are summarized in **Table 2**. For fibrous materials, these are comparatively well defined, combining geometry with biodurability, and the operative mechanism is frustrated phagocytosis: macrophages attempt but fail to engulf fibers exceeding their engulfment capacity. Rigidity is separable from length, and a diameter threshold has been adopted in European harmonized classification to distinguish rigid from entangled carbon nanofibers. Nominal geometry is nonetheless not sufficient, since thin nanofibers may entangle and lose fiber-like behavior, while aligned bundles of individually non-critical fibers may meet the criteria as secondary structures. Notably, lysosomal damage and NLRP3 activation are not exclusive to pathogenic fibers and have been reported across several carbon nanomaterial morphologies, so that inflammasome activation alone is an unreliable predictor of fiber-like pathogenicity; the selective extracellular release of lysosomal luminal proteins in the absence of lactate dehydrogenase release has been proposed as a more specific in vitro marker (65). For non-fibrous particles the determinants are less sharply defined and include size, shape, and dispersion state, with contradictory findings reported for shape (66). All of these properties depend on dose and cellular uptake, so that the same material may behave differently at different exposure levels.

**Table 2:** Material determinants associated with the magnitude and persistence of inflammasome activation.

Determinant	Value or direction	System/criterion	Ref.
Fiber length	≥ 5 µm	WHO critical fiber criterion	(65)
Respirable diameter	≤ 3 µm	WHO critical fiber criterion	(65)
Aspect ratio	≥ 3:1	WHO critical fiber criterion	(65)
Biodurability	Required	Part of the fiber pathogenicity paradigm; linked with morphology to fibrosis, lung cancer and mesothelioma	(65)
Macrophage engulfment capacity	10–20 µm (human)	Fibers exceeding macrophage dimensions may induce frustrated phagocytosis	(65)
Rigidity (as diameter)	>50 nm causes lysosomal damage; <20 nm does not	High-aspect-ratio materials of identical 2 µm length	(65)
Rigid vs entangled morphology	30 nm diameter threshold	Carbon nanofibers; diameter used to distinguish rigid from entangled morphologies	(65)
Particle size (non-fibrous)	Smaller more active than larger	Silver, iron oxide, titanium dioxide	(66)
Particle shape (non-fibrous)	Nanobelts, nanorods, octapods more active than spheres	Metal nanoparticles; contrary findings also reported	(66)
Dispersion state	Material-dependent; well-dispersed more active than poorly dispersed	Metal nanoparticles	(66)

Values for fibrous materials reflect established toxicological or regulatory criteria where applicable; for non-fibrous particles, only qualitative or material-dependent directional effects are summarized.

These same determinants suggest where formulation might intervene. Nanotechnology-based strategies to suppress NLRP3 activation in inflammatory disease target several upstream mechanisms, including reactive oxygen species, calcium flux, and mitochondrial dysfunction (67). Engineering a carrier so that it engages the inflammasome transiently and is then cleared, rather than avoiding engagement altogether, may therefore be a more realistic design objective than eliminating activation. It should be stated, however, that quantitative dose- or material-specific thresholds separating adjuvant-scale from pathological activation have not been established, and are unlikely to transfer directly between materials or exposure contexts: the molecular mechanisms by which particulates activate NLRP3 remain incompletely defined, and the absence of mechanistic insight into particle-biological interactions is recognized as the principal obstacle to predicting which exposures will resolve and which will persist (64).

### 3.3 Cellular responses of innate immunity

Once nanoparticles have been recognized by soluble sensors and pattern recognition receptors, the response is carried out by the cellular effectors of innate immunity. These populations engage nanomaterials through complementary mechanisms, ranging from uptake and antigen presentation to cytotoxicity, degranulation, and cytokine release. Although each cell type is considered separately below, their responses are interdependent and collectively determine the innate immune outcome (**Table 3**).

**Table 3:** Principal innate immune components and their interactions with nanoparticles.

Immune component	Role in NP encounter	Pathways activated	Effect in TME	Ref.
Macrophages (TAM)	Phagocytosis, polarization M1/M2	NF-κB, STAT3, PI3K-Akt- mTOR, metabolic reprogramming	M2→M1 repolarization: tumoricidal activity	(23, 24, 32)
Dendritic cells	Uptake, processing, Ag cross-presentation	TLRs, STING, MHC-I cross-presentation	DC maturation, CD8^+^ T cell priming	(7, 8, 70)
NK cells	Direct cytotoxicity, ADCC	NKG2D, DNAM-1, NCRs, CD16 (FcγRIIIa)	Direct tumor cell killing, ADCC	(72)
Neutrophils	Phagocytosis, NET formation	MPO, NE, PAD4, ROS-dependent NETosis pathways	Context-dependent pro or anti-tumoral effects, immune cell recruitment	(73)
Complement	Opsonization, immune activation	Classical, alternative, lectin pathway	Opsonization, CARPA, clearance, inflammation	(50, 51)
Mast cells	Degranulation, mediator release	FcϵRI signaling, TLR-associated pathways	Vascular modulation of TME, inflammatory amplification	(13, 74)

Entries summarize the principal reported interactions for each cell population and are not exhaustive; responses are formulation- and context-dependent.

#### 3.3.1 Macrophages and M1/M2 polarization

Macrophages occupy a central node in the nanoparticle–innate immunity axis. These highly plastic cells exhibit a continuum of polarization states, schematically classified as M1 (classically activated, pro-inflammatory and tumoricidal, characterized by the production of TNF-α, IL-12, and iNOS) and M2 (alternatively activated, anti-inflammatory and pro-reparative, characterized by the production of IL-10, TGF-β, and arginase-1) (23, 24, 32). The M2 compartment itself is heterogeneous and comprises at least three functionally distinct subsets: M2a (driven by IL-4/IL-13 and associated with pro-fibrotic activity), M2b (immune complex-driven and immunoregulatory), and M2c (driven by IL-10/TGF-β and associated with deactivation functions) (54). Underlying these phenotypes is a profound metabolic divergence: M1 macrophages rely predominantly on aerobic glycolysis and a disrupted Krebs cycle with succinate accumulation, whereas M2 macrophages preferentially utilize oxidative phosphorylation and fatty acid oxidation. This metabolic bifurcation provides pharmacological opportunities, including glycolytic inducers and OXPHOS inhibitors, that nanoparticles can potentially deliver locally to bias macrophage polarization (68, 69). Within the tumor microenvironment, tumor-associated macrophages (TAMs) predominantly display an M2-like phenotype, contributing to immunosuppression, angiogenesis, and tumor progression (23, 24). Lin et al. systematically reviewed how nanoparticle physicochemical properties modulate macrophage polarization: nanoparticle size influences polarization through lysosomal stress; nanoparticle morphology through membrane curvature and metabolic modulation; nanoparticle surface chemistry through membrane interactions; and nanoparticle rigidity through mechanosensitive ion channels (32). As a result, the capacity to repolarize TAMs from an M2-like toward an M1-like phenotype has emerged as one of the most actively pursued strategies in cancer immunotherapy, and multiple nanoparticle platforms have shown promising preclinical efficacy in this context (23, 24, 32).

#### 3.3.2 Dendritic cells and cross-presentation

DCs occupy a critical position as the principal antigen-presenting cells (APCs) bridging innate and adaptive immunity. This population is functionally heterogeneous: conventional type 1 DCs (cDC1) specialize in cross-presentation of exogenous antigens on MHC-I to CD8^+^ T cells and play a central role in antitumor immunity; conventional type 2 DCs (cDC2) primarily drive CD4^+^ helper T-cell responses; and plasmacytoid DCs (pDCs) are the principal producers of type I interferons (70). Each subset displays a distinct nanoparticle uptake profile, and formulations preferentially targeting cDC1 are of particular interest in oncology (70). Nanoparticle uptake by DCs, followed by antigen processing and cross-presentation of tumor antigens to cytotoxic CD8^+^ T lymphocytes, constitutes a key mechanism through which nanomedicine enhances antitumor immune responses (14, 15, 70). Mechanistically, cross-presentation proceeds via either a cytosolic pathway, in which internalized antigens escape from the phagosome, undergo proteasomal processing, and are loaded onto MHC-I via the TAP transporter, or a vacuolar pathway operating within endolysosomal compartments. Nanoparticles engineered to promote endosomal escape, particularly LNPs containing ionizable lipids, preferentially engage the cytosolic route, resulting in more efficient CD8^+^ T-cell priming. Recent studies further demonstrate that specific LNP formulations promote DC maturation through STING-dependent signaling, leading to expansion of migratory DC subpopulations and enhanced CD8^+^ T-cell responses compared with conventional protein adjuvants (14, 15). Han et al. developed LNPs containing substitutional adjuvant lipidoids that preserve high mRNA delivery efficiency while conferring TLR7/8 agonistic activity, thereby amplifying the innate immune component of mRNA-LNP vaccines (71).

#### 3.3.3 Natural killer cells

NK cells are innate lymphocytes capable of eliminating tumors and infected cells without prior antigenic sensitization through the balance of activating (NKG2D, DNAM-1, natural cytotoxicity receptors) and inhibitory (KIR, NKG2A) signals. Two complementary recognition modes govern their activity: “missing-self” recognition, in which target cells with downregulated MHC-I, a common feature of tumor and virally infected cells, evade inhibitory signaling and become susceptible to NK-cell-mediated killing; and “induced-self” recognition, in which stress-induced ligands (MICA, MICB, ULBPs) engage NKG2D on NK cells, providing a positive activation signal (72). Nanoparticle-based interventions can act on either side of this balance by reducing inhibitory restraint or amplifying stress-ligand expression on target cells. NK cells additionally mediate antibody-dependent cellular cytotoxicity (ADCC) through CD16 (FcγRIIIa), a mechanism that can be exploited by nanoparticle-delivered antibodies or antibody fragments. Nanoparticles can enhance NK-cell activity through several complementary mechanisms, including delivery of stimulatory cytokines (IL-15, IL-21), modulation of the NKG2D signaling pathway, and genetic engineering of NK cells to express chimeric antigen receptors (72). Murugan et al. reviewed five major areas in detail: nanoparticle-assisted immunomodulation, enhanced homing, RNAi delivery, genetic modulation, and NKG2D activation (72). NK cells are particularly attractive effectors for allogeneic off-the-shelf approaches because, unlike T cells, they do not induce graft-versus-host disease across HLA barriers. Consequently, nanoparticle-based strategies for NK-cell modulation represent a promising platform for universal cellular therapies.

#### 3.3.4 Neutrophils and extracellular traps

Neutrophils are the most abundant circulating leukocytes and constitute the first responders to sites of inflammation (73). Although their half-life in circulation is short, on the order of hours, their functional repertoire is remarkably broad and includes phagocytosis, degranulation, oxidative burst, cytokine release, and the formation of neutrophil extracellular traps (NETs). NETs are decondensed chromatin networks enriched in histones and antimicrobial granule proteins, including elastase, myeloperoxidase, and cathepsin G, which are expelled into the extracellular space to immobilize and eliminate pathogens. Two modes of NET release are currently recognized: “suicidal NETosis”, a lytic and NADPH-oxidase-dependent process culminating in neutrophil death over several hours; and “vital NETosis”, in which anuclear cytoplasts remain functionally active following chromatin release. Nanoparticles can trigger either mode and, in tumor settings, NET-mediated entrapment has been implicated in metastasis, adding a clinically relevant and therapeutically actionable dimension to neutrophil-nanoparticle interactions (73). Rezaei et al. reviewed nanomedical strategies for neutrophil targeting, highlighting how neutrophil tropism can be exploited to direct therapeutic agents toward pathological sites and how nanoparticle-mediated modulation of neutrophil function may be used to control inflammation (73). The therapeutic rationale is bidirectional: neutrophil-mimetic nanoparticles can exploit the cell’s intrinsic homing toward inflamed endothelium, whereas NET-disrupting formulations, including DNase-functionalized or histone-sequestering systems, can dismantle NETs already established within the tumor microenvironment.

#### 3.3.5 Mast cells

Mast cells are tissue-resident innate immune cells that contribute to both homeostatic and inflammatory processes. In nanomedicine, they are increasingly recognized as key mediators of hypersensitivity reactions, including complement activation-related pseudoallergy (CARPA), a phenomenon strongly associated with nanoparticle administration (50, 51). Nanoparticle-induced mast cell activation may occur through FcϵRI- and pattern recognition receptor-associated signaling pathways, leading to rapid degranulation and release of vasoactive mediators such as histamine, proteases, and pro-inflammatory cytokines (7, 74). These events can contribute to vascular modulation within the tumor microenvironment, influencing vascular permeability and immune cell recruitment. Importantly, mast cell responsiveness can be modulated by nanoparticle physicochemical properties, including surface chemistry, charge, and size, highlighting their relevance in both safety assessment and immunomodulatory design of nanomedicines (13, 74).

### 3.4 Trained immunity and nanoparticles

The recognition that the innate immune system possesses a form of memory, termed trained immunity, marked a substantial conceptual shift in immunology. Through epigenetic reprogramming (including histone modifications such as H3K4me3 and H3K27ac, as well as DNA methylation) and metabolic reprogramming (characterized by a shift toward aerobic glycolysis, activation of the pentose phosphate pathway, and disruption of the Krebs cycle with fumarate and mevalonate accumulation), monocytes, macrophages, and NK cells acquire an enhanced responsiveness to subsequent stimuli that can persist for weeks to months (20, 21). Canonical inducers of trained immunity, including BCG vaccination, β-glucan from fungal cell walls and oxidized LDL, share the capacity to engage a metabolic–epigenetic axis in which mevalonate-pathway intermediates regulate histone-modifying enzymes. Nanoparticles capable of reaching the bone marrow may act at an even earlier level by reprogramming hematopoietic progenitor cells themselves (so-called “central trained immunity”), thereby extending the duration of the response from weeks to months.

Nanoparticles can induce trained immunity through several mechanisms, including activation of the mTOR/Akt metabolic axis, epigenetic modulation via surface-associated patterns, persistent PRR stimulation, and modulation of cholesterol metabolism. Wang et al. described how specific nanostrategies enhance trained immunity in the vaccine setting by optimizing the physicochemical properties of nanocarriers to improve delivery to and uptake by innate immune cells (20). Teunissen et al. demonstrated the applicability of trained-immunity-targeting nanobiologics in cardiovascular disease, showing that HDL-mimetic nanoparticles modulate atherosclerotic inflammation through macrophage epigenetic reprogramming (21). This framework can be extended to oncology, where tumors may benefit from the induction of trained immunity, in contrast to atherosclerosis, which may benefit from its suppression. In this context, nanoparticle-induced trained immunity-particularly via metallic nanoparticles-remains an emerging but still underexplored frontier (22).

Detecting nanoparticle-induced trained immunity in humans requires biomarkers at three levels. In the bone marrow, BCG vaccination of healthy volunteers induces a persistent transcriptional program in the hematopoietic stem and progenitor compartment, regulated by hepatocyte nuclear factors 1a and 1b and conveyed epigenetically to circulating CD14+ monocytes (75). Sampling this compartment in humans is feasible, as sternal bone-marrow aspirates have been used to characterize persistent epigenetic alterations in CD34+ HSPCs and their myeloid progeny in patients with type 2 diabetes (76).

In peripheral blood, single-cell chromatin accessibility and transcriptomic profiling resolves vaccination-induced remodeling: seasonal influenza vaccination diminished H3K27ac and accessibility at AP-1 loci, whereas an adjuvanted formulation increased accessibility at interferon-regulatory-factor loci, with heterologous resistance to unrelated viruses (77). Transcriptomic profiling of monocytes before and after BCG vaccination, with secondary ex vivo stimulation, identified 75 genes whose response was altered and showed reorganization of co-expression modules, with systemic inflammation reduced rather than enhanced (78): training is not necessarily unidirectional, and a panel assuming a single direction of change would misclassify part of the response. At the functional level, ex vivo restimulation with an unrelated agonist remains a key operational readout of training, with IL-1β among the commonly measured biomarkers (79), alongside more accessible surrogates such as granulocyte counts and serum alpha-1 antitrypsin (75).

The relevant timescale is months rather than days. Enhanced monocyte cytokine responses following BCG vaccination persist for at least three months and are accompanied by increased H3K4me3 at the corresponding gene promoters (80), and the transcriptional signature transmitted from progenitors to circulating monocytes remains detectable three months after vaccination (75). Because circulating monocytes survive only days, persistence over such intervals is consistent with, and may reflect, reprogramming at the progenitor level rather than persistence of the original mature-cell population, and dictates a sampling schedule extending well beyond the acute post-administration window.

A study designed to detect nanoparticle-induced training in humans would pair longitudinal peripheral sampling with functional and epigenomic readouts, reserving marrow sampling for settings where it is already clinically indicated; frameworks for translating trained-immunity endpoints into drug development have recently been outlined (81). A prospective design would include baseline sampling, early post-treatment sampling to capture acute activation, and follow-up at one to three months or longer to distinguish transient inflammation from persistent reprogramming, with an unrelated ex vivo stimulus to assess functional training.

Two obstacles are specific to nanomaterials. Innate memory can be induced by particles that produce no direct inflammatory activation and would therefore pass conventional immunotoxicity testing, which argues for including memory-related endpoints in immunosafety assessment rather than treating them as a question of efficacy (82). And in human primary monocytes exposed to gold and iron oxide nanoparticles, memory profiles depended on size, shape and composition but were above all strongly donor-dependent (83); physicochemical determinants of innate training by particulates have been reviewed separately (84). No validated biomarker is currently available, and inter-individual variability may prove the principal obstacle to establishing one.

### 3.5 Immunogenicity: desired and undesired

Nanoparticle immunogenicity is not an intrinsically positive or negative property; rather, its consequences depend on the therapeutic context, which is a unifying principle of this review (**Figure 2**). In drug delivery, immunogenicity is generally undesirable because it can accelerate clearance, trigger complement activation-related pseudoallergy (CARPA) and promote the formation of anti-PEG antibodies that may compromise pharmacokinetics and efficacy upon re-administration through the “accelerated blood clearance” (ABC) phenomenon (49–51).

**Figure 2:**
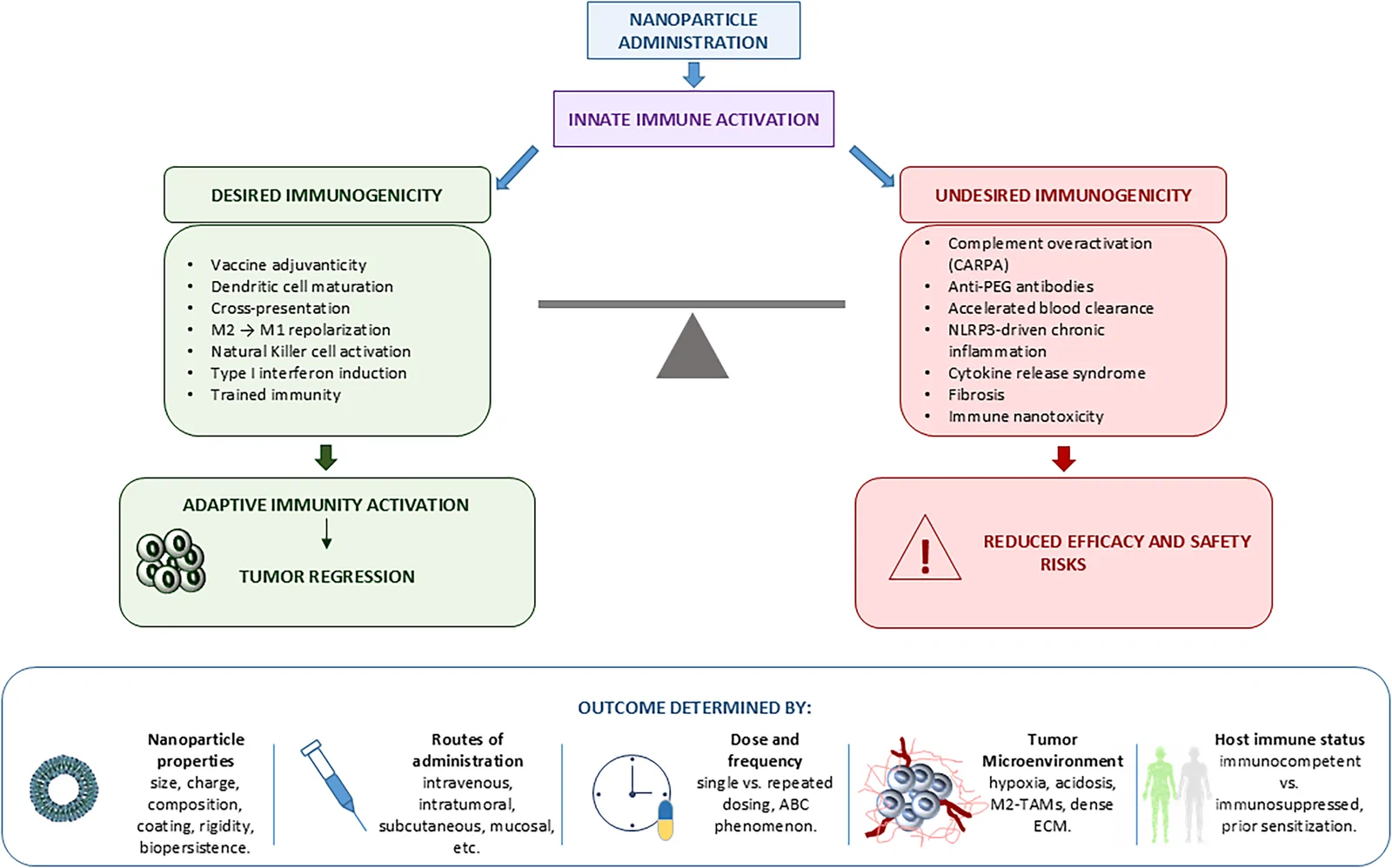
The dual nature of nanoparticle immunogenicity: a balance between therapeutic benefit and safety risk. Innate immune activation following nanoparticle administration can be steered toward either of two opposite outcomes. When harnessed deliberately (left), it produces desired immunogenicity — vaccine adjuvanticity, dendritic cell maturation, cross-presentation, M2-to-M1 macrophage repolarization, natural killer cell activation, type I interferon induction, and trained immunity — which drives adaptive immune activation and, ultimately, tumor regression. When it escapes control (right), the same activation results in undesired immunogenicity, including complement overactivation and complement activation-related pseudoallergy (CARPA), pre-existing and treatment-induced anti-PEG antibody responses, accelerated blood clearance, NLRP3-driven chronic inflammation, cytokine release syndrome, fibrosis, and immune nanotoxicity, all of which compromise both efficacy and safety. Which side of the balance prevails is not an intrinsic property of the material but is determined by nanoparticle physicochemical features (size, charge, composition, coating, rigidity, biopersistence), route of administration, dose and dosing frequency, the state of the tumor microenvironment, and the immune status of the host.

The ABC phenomenon is therefore a relevant consideration, although its clinical incidence remains incompletely defined. Quantitative data on anti-PEG seroprevalence and on susceptibility to ABC are summarized in **Table 4**. Three points emerge. Pre-existing antibodies are relatively common, although reported seroprevalence varies substantially across studies, in part because of differences in assay design, antigen presentation and analytical thresholds (85–87). They are also longstanding, predating the recent expansion of PEGylated nanomedicines, although anti-PEG antibody levels may be influenced by previous exposure to PEG-containing products, including PEG-containing therapeutics and mRNA-LNP vaccines (88, 89).

**Table 4:** Anti-PEG antibody prevalence and susceptibility to accelerated blood clearance.

Parameter	Value	System	Qualification	Ref.
Seroprevalence, general population	44.3%–72%	Human plasma/sera, different assays	Substantial analytical and population heterogeneity	(85–87)
Seroprevalence, earlier/less-sensitive assays	<1%–44%	Human sera	Strongly assay- and threshold-dependent	(86)
Anti-PEG IgG >500 ng/mL	~7% of samples	Human sera	Most positive individuals have low titers	(86)
Anti-PEG IgM >500 ng/mL	~1% of samples	Human sera	—	(86)
IgG2 as share of anti-PEG IgG	~83%	Human sera	IgG2 predominance compatible with T-cell-independent induction; co-occurring IgG1 (~16%) argues against a purely T-independent mechanism	(86)
Sera banked 1970–1999	56% positive	Archival human sera	Predates widespread use of PEGylated nanomedicines	(86)
Predictors of ABC (correlation)	r = 0.671 drug-release surrogate; 0.509 IgM; 0.115 IgG	Rats exposed to PEG excipients	Functional assay showed stronger association with ABC than antibody titers	(91)
Accelerated drug release >0.2 µg/mL	94/1,764 (5.33%)	Human plasma	Assay-based susceptibility, not observed ABC incidence	(91)
Above threshold at clinical PEG-liposomal doxorubicin dose	5/1,764 (0.28%)	Human plasma	Thresholds extrapolated from rat data; not a clinical ABC incidence estimate	(91)
Effect of dose within one platform	1900-fold vs 3.5-fold reduction in exposure	Rats, low vs ten-fold higher dose	Exposure/dose is an important determinant of ABC magnitude	(91)
Anti-PEG titers vs circulating mRNA-LNP	No clear association with baseline titers	19 human subjects, i.m. booster	Biodistribution study rather than direct ABC study; post-vaccination antibody increases associated with LNP detected in blood	(90)
ABC without detectable anti-PEG IgM/IgG	Reported	PEGylated cerasomes in rats	ABC occurred without detectable antibody elevation; complement activation was also observed	(92)

Values are reported as published; the qualification column indicates the interpretive limitations of each measure. Assay-based estimates of susceptibility should not be interpreted as clinically observed ABC incidence.

Prevalence should not be equated with a comparable incidence of ABC. The magnitude and clinical relevance of the effect depend on antibody concentration, particle composition, dosing regimen and route, and the evidence differs sharply between platforms. For intramuscular mRNA-LNPs, quantitative evidence directly linking pre-existing anti-PEG antibodies to accelerated clearance in humans remains limited, and human pharmacokinetic data do not currently establish a simple relationship between pre-existing anti-PEG antibodies and LNP disposition (90). For intravenously administered PEGylated liposomes, a functional plasma assay predicted ABC more closely than antibody titers and placed the proportion of susceptible individuals at a clinically relevant dose below 1%; these are assay-based estimates extrapolated from rat thresholds, not observed clinical incidence (91). Neither dose nor antibody titer is sufficient on its own: exposure within a single platform varied by three orders of magnitude with dose, and pronounced ABC has been reported in the absence of detectable anti-PEG IgM but with significant complement activation (92).

In the context of vaccine and cancer immunotherapy applications, the same immunostimulatory properties can become therapeutically advantageous: the ability of LNPs to function as intrinsic adjuvants is now recognized as a defining feature of the mRNA platform (13, 16). Verbeke et al. analyzed the innate mechanisms of mRNA-LNP vaccines and showed that the LNP carrier can act as a potent adjuvant independently of the mRNA cargo (16). This dualism, in which the same property (innate immune activation) can be detrimental in one context yet therapeutically valuable in another, represents the central design challenge of rational nanomedicine and provides the conceptual foundation for the applications discussed throughout this review.

### 3.6 Immune nanotoxicology

Immune nanotoxicology studies the adverse effects of nanoparticles on the immune system. Beyond CARPA, the spectrum of concern includes chronic inflammation sustained by persistent macrophage and inflammasome activation; immunosuppression caused by APC depletion; and disruption of pulmonary immune homeostasis following inhalation exposure (7, 8, 61, 93). The organ distribution of adverse effects broadly parallels the biodistribution of nanoparticle accumulation. The liver, as the dominant site of nanoparticle sequestration, is particularly vulnerable to Kupffer cell-mediated inflammation and, in the long term, to progressive fibrosis. The spleen may develop granulomatous alterations after repeated exposure. The lung, when the route is inhalation, presents a specific set of risks, including alveolar macrophage overload, frustrated phagocytosis of high-aspect-ratio nanomaterials, and, in the case of biopersistent fibers, chronic fibrotic remodeling reminiscent of asbestos-associated pathology. The kidney, by contrast, is generally protected by glomerular filtration because only particles below the hydrodynamic threshold of approximately 5.5 nm noted above can be filtered; above this threshold, renal accumulation requires specific physicochemical or biological transport mechanisms but, when it occurs, can promote tubular inflammation.

Lipopolysaccharide (LPS) contamination of nanoparticle preparations remains a critical confounder in immunotoxicological studies, a point specifically emphasized by Boraschi and Decuzzi (7). Even low levels of LPS can contribute substantially to cytokine responses and generate false-positive signals of nanoparticle immunostimulation if endotoxin contamination is not adequately controlled (7, 94). Moreover, nanoparticle suspensions may interfere with conventional Limulus amebocyte lysate (LAL) assays: no single LAL format proved optimal across the nanoformulations tested, and the macrophage activation test used as a complementary approach may itself be unsuitable for formulations carrying cytotoxic drugs. Endotoxin measurements should therefore be performed using methods appropriately evaluated for the specific nanoformulation (94, 95). Accordingly, rigorous endotoxin control is important when assessing claims of intrinsic nanoparticle immunogenicity and should, where feasible, include complementary approaches such as polymyxin B neutralization, heat inactivation, or, where biologically appropriate, testing in TLR4-deficient systems (7, 94).

de Braganca et al. reviewed adverse immunological responses to non-viral delivery systems in the lung, analyzing the relationship between physicochemical properties and interactions with alveolar macrophages and DCs (93).

Attributing an observed innate response specifically to the nanoparticle itself therefore requires distinguishing among three possible sources: (i) the carrier or nanomaterial, (ii) the cargo it delivers, including RNA-, DNA-, or mtDNA-dependent signaling, and (iii) contaminants introduced during synthesis or handling, particularly endotoxin/LPS. Each requires appropriate controls to support causal attribution. Carrier-driven activation is supported by comparison with an empty carrier prepared using the same procedure, whereas cargo-driven activation is supported by comparison with the free cargo at a matched dose. Evidence against contaminant-driven activation requires endotoxin quantification using methods validated for the specific nanomaterial (94, 95), together with complementary controls where feasible. When the proposed mechanism is receptor-specific, genetic or pharmacological blockade of the relevant receptor or pathway provides stronger evidence for causal involvement. In the absence of appropriate carrier, cargo, or contaminant controls, an observed innate response should not be attributed specifically to the nanoparticle material itself and is better described as uncertain or context-dependent.

These distinctions are applied throughout the class-specific sections that follow. In several of the systems discussed, receptor engagement attributed to a platform is in fact attributable to a co-delivered agent rather than to the material, and this is stated where it applies.

A further distinction, which cuts across all three sources, concerns what an immune response to the carrier actually changes. After intramuscular administration in mice, circulating anti-PEG IgM had only a minor effect on translation and distribution within the injected muscle and did not increase the total hepatic accumulation of mRNA-LNPs; hepatic protein translation was nevertheless markedly diminished, because the particles were delivered primarily to Kupffer cells rather than to hepatocytes, with antibody binding and subsequent complement activation suppressing translation in the liver. Repeated intramuscular immunization with SARS-CoV-2 Spike mRNA-LNPs still elicited a robust humoral response (96). Antibody recognition may therefore redirect the cellular fate of the carrier, and with it the functional output of its cargo, without altering the amount of material reaching the organ: a dissociation between biodistribution and function that measurements of tissue accumulation alone would not detect. The route of complement activation is itself variable between individuals: in human plasma samples showing accelerated drug release from PEGylated liposomes, ion-chelation experiments indicated that the pathway engaged differed from donor to donor (91).

## 4 Class-specific immunological profiles of nanoparticles

This section builds on the general mechanisms discussed in Section 3 by integrating the compositional and structural characteristics of each of the six nanoparticle classes, whose immunological profiles are summarized in **Table 5**.

**Table 5:** Comparative immunological profiles of the six nanoparticle classes. Grading criteria and their limitations are given in the note following the table.

NP class	PRR/innate sensor engagement	Complement activation	Cytokine/polarization signature	Evidence base	Oncological application/development status	Ref.
Liposomes/LNP	TLR4 reported for certain ionizable lipids; TLR7/8 for mRNA cargo; TLR9 and cGAS–STING for some nucleic-acid cargo. Carrier and cargo may both contribute, depending on formulation	Context-dependent; high activation has been documented for some formulations, including CARPA associated with PEGylated systems and anti-PEG IgM-mediated complement activation	Type I IFN induction; intrinsic adjuvanticity can occur independently of cargo; CD8^+^ T-cell expansion	Human clinical and preclinical evidence	mRNA-based cancer vaccines; cytokine and drug delivery; liposomal doxorubicin (Doxil) is clinically approved in oncology	(14, 49–51, 53, 97–101, 107)
Exosomes/PVLN	No single dominant PRR has been established; innate effects are cargo-, source- and formulation-dependent	Context- and route-dependent; mammalian exosomes can express CD55/CD59, limiting complement-mediated damage; PVLN responses are formulation- and route-dependent	Modulation of inflammatory cytokine output; PVLN can suppress macrophage activation; engineered systems can promote M1-like repolarization	Early clinical (Phase I) for selected exosome-based approaches; preclinical in vivo for PVLN	TAM reprogramming; miRNA and bioactive-molecule delivery	(108–113 )
Polymeric nanocapsules	No dominant PRR established; MPS recognition through opsonization, with downstream NF-κB and PI3K–Akt–mTOR signaling reported for selected formulations	Formulation-dependent; PEGylated PLGA has been reported to reduce complement activation and neutrophil recruitment relative to non-PEGylated PLGA	TNF-α, IL-6, IL-1β modulation; M1-like repolarization; immunometabolic effects	Preclinical in vivo	Multidrug local release; antigen and immune-checkpoint inhibitor delivery	(40, 42, 43, 114, 115)
AuNP/AuNR	cGAS–STING and TLR3 activation reported in multifunctional systems, often attributable to co-delivered agonists rather than to gold itself; recognition also influenced by protein corona	Context-dependent; bare AuNPs can strongly activate complement, whereas protein corona formation and PEGylation can attenuate activation in specific systems	ICD-associated DAMP release (CRT, HMGB1, ATP); type I IFN-associated responses in multifunctional systems; M1-like repolarization through engineered IgG corona	Preclinical in vivo (murine tumor models)	PTT + ICD; photo-immunotherapy	(41, 44, 116–121)
Copper-based nanoparticles and nanoplatforms	Indirect cGAS–STING activation can occur through mitochondrial stress and mtDNA release; direct STING activation in therapeutic systems may instead result from co-delivered STING agonists such as MSA-2. ROS-driven inflammasome-related signaling has also been reported	Documented in CuS systems; C5a/C5aR signaling was associated with cuproptosis resistance through ATP7B upregulation	Cuproptosis-associated ICD/DAMP release; DC maturation; cytotoxic T-cell activation	Preclinical in vivo (murine tumor models), with mechanistic support from non-oncological in vitro studies	Cuproptosis + immunotherapy; pH-responsive and stimulus-responsive cuproptosis platforms	(45–48, 123, 125, 126)
Phytochemical nanoformulations	TLR4 inhibition reported for fucoidan; no consistent activating PRR engagement has been established across the class	Carrier- and size-dependent in the studied liposomal systems: targeted and PEGylated liposomes increased iC3b above buffer levels, while SC5b-9 and iC3b correlated with vesicle diameter rather than clearly with the phytochemical payload	NF-κB/STAT3 inhibition; M2→M1-like shift; reduced VEGF/HIF-1α and PD-L1	Preclinical in vivo and in vitro studies	TAM repolarization; anti-angiogenic and immunomodulatory therapy	(113, 127, 130)

Comparisons are semi-quantitative and should be interpreted as evidence-weighted qualitative profiles rather than as directly comparable quantitative measures. Complement activation is graded as low, moderate, or high only where sufficient evidence permits; grading is based on the available evidence, including reported CARPA, nanoparticle-reactive antibody responses, complement deposition or consumption assays, and related *in vitro* or *in vivo* findings. These profiles are formulation-, assay-, route-, and context-dependent and may vary substantially between recipients. “Evidence base” indicates the highest level of evidence supporting the reported profile as a whole — human clinical data, preclinical *in vivo* studies, or *in vitro* work — and does not imply that every entry in the row derives from that level. Where a mechanistic association is supported primarily by a non-oncological experimental model, this is indicated in the text and should not be interpreted as direct evidence of an oncological therapeutic effect. Where a mechanistic association is supported primarily by a specific cargo, surface coating, protein corona, or co-delivered immunomodulator, this dependency is indicated explicitly rather than attributed to the nanoparticle class as an intrinsic property.

The classification adopted here is functional rather than strictly taxonomic: systems are grouped according to the component that predominantly governs their innate immune engagement rather than solely according to material chemistry, and the six categories are therefore neither equivalent in scope nor mutually exclusive. A curcumin-loaded liposome, for instance, belongs both to the lipid-based platforms of Section 4.1 and to the phytochemical formulations of Section 4.6; which section is the more informative depends on whether the innate response is driven primarily by the carrier or by the payload. Where a formulation could reasonably be assigned to more than one class, it is discussed under the category corresponding to its principal immunological driver and cross-referenced, and the overlap is acknowledged rather than resolved by arbitrary assignment.

### 4.1 Liposomes and lipid nanoparticles

Liposomes, phospholipid bilayer vesicles typically ranging from 50 to 200 nm, represent the longest-standing nanocarriers in clinical use, with more than three decades of therapeutic application (97, 98). Next-generation lipid nanoparticles (LNPs), composed of ionizable lipids, helper phospholipids, cholesterol, and PEG-lipids, reshaped the field through their central role in mRNA COVID-19 vaccines (5, 13, 16, 98). Beyond their function as delivery vehicles, LNPs can themselves modulate innate immune responses, making it important to distinguish carrier-driven immunostimulation from effects attributable to the nucleic-acid cargo or to formulation contaminants.

Materials such as ALC-0315, SM-102, and newer adjuvant lipidoids have been investigated as potential contributors to innate immune activation and, depending on the formulation, have been reported to engage TLR4-associated inflammatory signaling and/or STING-dependent dendritic-cell activation. Tursi et al. showed that DNA-LNP formulations induce STING-dependent expansion of migratory DC subpopulations, resulting in enhanced CD8^+^ T-cell responses and long-lasting adaptive immunity compared with conventional protein-adjuvant formulations (14). Similarly, Oyama et al. designed an ionizable lipid built on a vitamin E scaffold that strengthens both cytotoxic T-cell responses and type I interferon-dependent innate activation (99). These observations indicate that the immunological activity of LNPs can depend on the lipid composition itself, although the contribution of the cargo and other formulation components must be experimentally controlled.

The PEG-lipid component, by contrast, is the principal liability: on repeated administration, it can elicit anti-PEG antibodies, leading to accelerated blood clearance (the ABC phenomenon) and a heightened risk of complement activation-related pseudoallergy (CARPA), a problem that is particularly relevant for therapies requiring chronic or repeated dosing (49–51). The prevalence of pre-existing anti-PEG antibodies, their relationship with ABC, and strategies to mitigate PEG-associated immunogenicity are addressed in the sections on immunogenicity and on clinical translation.

Beyond antibody- and complement-mediated effects, the adjuvant properties of LNPs are not confined to the mRNA cargo. Dai et al. demonstrated that LNPs possess intrinsic adjuvant activity in protein vaccines as well, acting through TLR9- and type I interferon-dependent mechanisms (100). Similarly, Zhivaki et al. showed that mRNA encoding a constitutively active cGAS mutant (cGASΔ;N), co-formulated within LNPs, behaves as a “catalytic adjuvant” that amplifies CD8^+^ T-cell responses (101). Together, these studies support the view that innate immune activation is not necessarily a consequence of the nucleic-acid cargo alone, and that the carrier can provide an independent immunological signal.

Not every immunological consequence is necessarily beneficial, however. The same innate immune activation that can be exploited for adjuvant activity may, if excessive or dysregulated, contribute to undesirable inflammatory responses. Myocarditis and pericarditis have been identified as rare adverse events following mRNA COVID-19 vaccination, occurring predominantly in young males after the second dose. A systematic review of cohort, registry and surveillance data found these cases to be generally mild and self-limited, with most patients recovering without complication, whereas myocarditis following SARS-CoV-2 infection was consistently associated with more severe outcomes including higher rates of hospitalization and mortality; the benefit–risk profile of mRNA vaccination was judged to remain strongly favorable (102). Reported incidence figures are of the order of 19.7 cases per million doses, against 2.76 cases per thousand infections (103). The underlying biological mechanism has not been established, and the European Medicines Agency has noted that the available research data remain scarce and that no clear understanding of the responsible mechanisms has yet emerged (104).

Within this broader framework, recent studies have begun to identify receptors that may sense ionizable lipids. Chaudhary et al. showed that blockade of TLR4 with TAK-242, or neutralization of CD1d, increased anti-PEG IgM production in ex vivo splenocyte cultures and in mice, indicating that inflammatory signaling downstream of these receptors suppresses the anti-PEG IgM response; consistently, lipids with stronger predicted receptor interactions retained delivery efficacy on repeat dosing, whereas those with weaker interactions lost more than 100-fold of their potency by the fifth dose (105). Binding was inferred from molecular docking rather than measured, and the calculated energies served as a predictive screen rather than as evidence of receptor occupancy.

In a human monocytic reporter line, LNPs formulated with ALC-0315 or SM-102 activated NF-κB and IRF predominantly through TLR4 and MyD88, confirmed with knockout lines; the response was abolished when the ionizable lipid was omitted, was comparable for empty and mRNA-containing LNPs, and was obtained with endotoxin below the detection limit (106). These controls support attribution to the ionizable lipid carrier rather than to the cargo or to endotoxin in this system, without establishing a universal property of ionizable lipids. The dependence is not absolute: a TLR4-knockout model retained vaccine responsiveness, indicating that other pathways compensate in vivo (106). Carrier-driven TLR4-dependent sensing is therefore supported in defined systems, and the same evidence illustrates the limits that should apply when assigning a specific receptor as the causal trigger.

Against this background, Glossmann proposed that TLR4 activation by ionizable lipids in cardiac microvascular endothelial cells might represent one initiating event, potentially amplified by spike-protein-mediated trained immunity following subsequent doses (107). The hypothesis extends observations on TLR4-dependent sensing of ionizable LNPs, obtained in monocytic cell lines in vitro, to a different cellular compartment and to a specific pathological outcome. Direct evidence for TLR4 activation by vaccine ionizable lipids in cardiac microvascular endothelial cells, or for a causal link between this pathway and myocardial injury, is currently lacking. The pathway should therefore be regarded as biologically plausible but unconfirmed, and considered alongside other proposed mechanisms including adaptive immune dysregulation, molecular mimicry, endothelial responses and host-specific susceptibility.

### 4.2 Exosomes and plant-derived vesicle-like nanoparticles

Exosomes (30–150 nm) are extracellular vesicles released by virtually all eukaryotic cells that mediate intercellular communication through the transfer of proteins, lipids, mRNA, and miRNA. In the tumor setting, they play a dual role: tumor-derived exosomes (TDEs) promote immunosuppression by delivering PD-L1, TGF-β, and immunosuppressive miRNAs, yet the same vesicles can be re-engineered into immunotherapeutic platforms (108, 109). Lu et al. reviewed this dual role in T-cell-based immunotherapy, contrasting the immunosuppressive functions of TME-derived exosomes against their therapeutic potential (109). On the engineering side, Wei et al. developed multifunctional exosomes loaded with Fe_3_O_4_ nanoparticles and the photothermal agent indocyanine green (ICG), leveraging intratumoral bacteria as antitumor effectors by combining ferroptosis, photothermal therapy, and CD47 blockade to polarize tumor-associated macrophages toward a tumoricidal M1-like phenotype (110). Beyond their cargo and therapeutic potential, exosomes also possess active mechanisms of immune evasion: they carry the glycosylphosphatidylinositol (GPI)-anchored complement regulators CD55 and CD59 on their surface, and antibody blockade of either increases C3b deposition and vesicle lysis, demonstrating that complement protection can be mediated by specific surface regulators rather than by vesicle composition alone (111).

Plant-derived vesicle-like nanoparticles (PVLNs) represent an emerging frontier. Wang et al. reviewed their biogenesis, composition, and physicochemical properties, describing immunomodulatory effects that span both innate immunity (including suppression of macrophage activation and modulation of inflammatory cytokine production) and adaptive immunity, through regulation of T-helper-cell responses (108). PVLNs are particularly attractive because they combine several advantageous features rarely found in a single delivery platform: excellent biocompatibility, the capacity for cross-species communication, high drug-loading potential, and scalable low-cost production. Unlike mammalian exosomes, PVLNs should not be assumed to possess intrinsically low complement reactivity. Reviewing the available safety data, Chen and Xiao report that intravenous administration of PVLNs can trigger complement activation, immune responses, and hepatorenal toxicity, and that these risks are not completely eliminated by surface engineering, whereas oral administration may reduce the immunogenic burden through gastrointestinal degradation. On this basis, they advocate prioritizing the oral route and caution against intravenous administration pending more comprehensive preclinical safety assessment (112). The low immunogenicity commonly attributed to plant-derived vesicles may therefore depend, at least in part, on the route of administration rather than representing an intrinsic and route-independent property of the material. By comparison, complement protection is better characterized in mammalian exosomes, in which GPI-anchored regulators such as CD55 and CD59 contribute to complement evasion, whereas corresponding mechanisms have not yet been established for plant-derived vesicles. Their convergence with phytochemicals offers the prospect of “natural phytonanovectors” for immunomodulation (108, 113).

### 4.3 Polymeric nanocapsules

Polymeric systems based on PLGA, PVA, chitosan, PCL (Polycaprolactone), and PEG offer versatile platforms with tunable release kinetics, biodegradability, and well-documented biocompatibility. Their interaction with the innate immune system depends strongly on physicochemical characteristics, including composition, size, surface charge, hydrophobicity, and surface functionalization (42, 43). Upon systemic administration, these nanoparticles are primarily recognized by cells of the mononuclear phagocyte system, particularly macrophages and dendritic cells, which represent the first immunological barrier and key determinants of their biodistribution and immunological fate. Kaur et al. reviewed the application of polymer-based nanoformulations in cancer immunotherapy, showing how FDA-approved polymers can be engineered to deliver tumor antigens, immune checkpoint inhibitors, cytokines, and small immunomodulatory molecules directly to the tumor microenvironment (43). Beyond their role as inert carriers, polymeric nanocapsules can actively modulate innate immune signaling pathways. Recent evidence indicates that they can influence macrophage polarization and dendritic cell activation through the regulation of NF-κB, PI3K-Akt-mTOR signaling, and metabolic reprogramming, thereby linking material properties with immune phenotype outcomes (114). Zhang et al. further demonstrated that polymeric nanoparticles could exert anti-inflammatory or pro-resolving effects depending on their design parameters, modulating cytokine production (e.g., TNF-α, IL-6, IL-1β) and reshaping macrophage inflammatory programs through coordinated immunometabolic rewiring (114). Among these parameters, surface chemistry can be particularly important in determining the innate response to the carrier itself. In contrast to the complement-activating effects associated with PEG-lipid-containing LNPs described above, PEGylated PLGA nanoparticles elicit lower complement activation and reduced neutrophil recruitment at the injection site than their non-PEGylated counterparts, consistent with a less inflammatory local microenvironment (115).

The immunological profile of polymeric nanocapsules can be further tailored through the control of particle architecture and mechanical properties. Key et al. demonstrated that soft discoidal polymeric nanoconstructs exhibit reduced macrophage uptake and prolonged vascular persistence compared with conventional spherical nanoparticles, highlighting the importance of geometry and deformability in regulating nanoparticle-immune cell interactions (40). These observations underscore a broader principle in nanomedicine: innate immune recognition is governed not only by chemical composition but also by physical design parameters that influence opsonization, phagocytosis, and biodistribution.

In the context of cancer immunotherapy, polymeric nanocapsules are increasingly being developed as multifunctional platforms capable of combining drug delivery with immune modulation. By controlling the spatial and temporal release of immunomodulatory agents, these systems can enhance dendritic cell activation, promote macrophage repolarization toward an M1-like phenotype, and improve the recruitment and activation of cytotoxic lymphocytes within the tumor microenvironment (43, 114).

### 4.4 Gold nanoparticles and nanorods (AuNP/AuNR)

Gold nanoparticles (AuNPs, typically 5–100 nm) and gold nanorods (AuNRs, aspect ratio 2-10) possess plasmonic properties that enable them to absorb near-infrared light (NIR, 650–1350 nm) and convert it into heat, forming the physical basis of photothermal therapy (PTT) (41, 44, 116). Their interaction with the innate immune system is multifaceted and depends on both their physicochemical properties and the combinatorial therapeutic context.

A first level of interaction involves the protein corona, which can be engineered on gold surfaces to actively modulate immune responses rather than merely influencing biodistribution. Park et al. demonstrated that a designed IgG-enriched nanocorona on PEGylated gold nanostars reprograms tumor-associated macrophages toward an M1-like phenotype through structural remodeling of adsorbed IgG, particularly through alterations in β-sheet and α-helix content, thereby synergizing with PTT to induce durable antitumor immunity and vaccine-like effects (117). The corona also governs complement recognition. Uncoated gold nanomaterials of three distinct morphologies (spheres, stars and rods) activate the classical, alternative and lectin pathways simultaneously in human serum, with formation of the terminal SC5b-9 complex and consequent complement-mediated uptake by human U937 promonocytic cells (118). Pre-formation of a protein corona markedly attenuated complement activation by AuNPs, irrespective of the tested particle size and surface charge, suggesting that complement recognition is strongly influenced by the nanoparticle–protein interface rather than by the gold core alone (119).

A second mechanism is linked to photothermal-induced immunogenic cell death (ICD), which promotes the release of danger-associated molecular patterns (DAMPs) such as calreticulin, high mobility group box 1 (HMGB1), and ATP, leading to dendritic cell maturation and cytotoxic T-cell activation. Cheng et al. developed a biomimetic gold nanorod-core/mesoporous MnO_2_-shell nanoagonist (m@AMCR) loaded with chlorin e6 and rapamycin, enabling synergistic photothermal and photodynamic (PTT/PDT) effects together with cGAS–STING pathway activation driven by Mn²^+^ release rather than by the gold component itself (44). Similarly, Huang et al. designed a macrophage membrane-coated AuNR platform (GNR-SNO@MMT), integrating thermosensitive nitric oxide donors, which, upon NIR-II irradiation, achieved potent tumor inhibition and enhanced CD8^+^ T-cell infiltration within the tumor microenvironment (116).

A third strategy involves multimodal combination therapies, where gold nanostructures serve as photothermal hubs integrated with immunomodulatory or epigenetic agents. Sun et al. developed nano-golden adjuvant polymersomes with gold nanoclusters embedded in the membrane and poly(I:C), a synthetic double-stranded RNA analog, encapsulated in the aqueous core; a single irradiation session following intratumoral injection achieved an 83% cure rate in murine LLC lung tumor models, rising to 100% complete regression when combined with cRGD functionalization and anti-CTLA-4 blockade, together with systemic antitumor immunity and abscopal effects (120). The contribution of TLR3 activation in this system is attributable to the co-delivered poly(I:C), rather than to the gold nanoclusters themselves. Gao et al. further extended this concept by integrating AuNRs with epigenetic inhibitors (5-azacitidine and mocetinostat), reprogramming the MYC/type I interferon axis and inducing durable immune memory responses (121).

Collectively, these studies highlight that gold-based nanomaterials act not only as photothermal transducers but also as active immunological modulators, capable of engaging macrophages, dendritic cells, and cytotoxic lymphocytes through interconnected pathways including antigen release, innate immune activation, and epigenetic reprogramming. However, several innate immune effects attributed to gold-based platforms are formulation-specific and may depend on co-delivered immunostimulatory agents or on the protein corona rather than on the gold core itself.

### 4.5 Copper-based nanoparticles and nanoplatforms

The discovery of cuproptosis by Tsvetkov and colleagues opened a new chapter in metal-based cancer therapy (45, 122). Cuproptosis is a regulated form of cell death triggered by intracellular copper accumulation, leading to aggregation of lipoylated tricarboxylic acid (TCA) cycle proteins, particularly dihydrolipoamide S-acetyltransferase (DLAT), and destabilization of iron–sulfur ([Fe–S]) cluster proteins, resulting in mitochondrial proteotoxic stress and impaired mitochondrial respiration and metabolic homeostasis (45, 46, 48, 122). What makes copper-based cytotoxicity particularly relevant in oncology is its mechanistic distinction from classical cell death pathways, including apoptosis, necroptosis, ferroptosis, and pyroptosis, thereby potentially offering an alternative strategy to overcome tumor resistance to conventional cell-death mechanisms (45, 46, 48).

Beyond cuproptosis, copper-based nanoparticles and nanoplatforms exploit the redox-active nature of copper to induce oxidative stress, mitochondrial dysfunction, and lysosomal damage, which can contribute to tumor cell killing. The ability of copper to cycle between Cu^+^ and Cu²^+^ states can promote ROS generation, which amplifies cellular stress and may promote immunogenic tumor cell death (45, 46). Complement engagement is not incidental in these systems. Copper sulfide nanoparticles activate the complement cascade, and C5a/C5aR signaling promotes resistance to cuproptosis by upregulating the copper exporter ATP7B through the Wnt/β-catenin pathway. Pharmacological blockade of C5aR reduces ATP7B expression, restores sensitivity to cuproptosis, and enhances the antitumor efficacy of CuS nanoparticles (123). Thus, complement activation may represent not only a potential limitation of copper-based nanotherapy but also a therapeutically actionable pathway.

Its connection to innate immunity is mediated through multiple converging pathways. In tumor models, copper-based nanotherapeutic strategies capable of inducing cuproptosis have been shown to elicit immunogenic cell death (ICD), characterized by calreticulin (CRT) exposure and the extracellular release of HMGB1 and ATP, thereby promoting dendritic-cell maturation and subsequent T-cell-mediated antitumor responses (45, 46, 124). In addition, copper-mediated mitochondrial stress and oxidative damage can promote the cytosolic escape of mitochondrial DNA (mtDNA) and downstream innate immune sensing. In copper-overloaded C8-D1A astrocytes, mitochondrial ROS accumulation drove mtDNA release into the cytosol, activating cGAS–STING and promoting a direct STING–NLRP3 interaction that assembled the inflammasome and induced pyroptosis; depletion of mitochondrial ROS reduced mtDNA leakage, and pharmacological inhibition of STING reversed pyroptosis, establishing the causal chain (125). Although demonstrated in a neurotoxicological rather than an oncological setting, and with ionic rather than nanoparticulate copper, these findings provide a mechanistic basis for linking copper-induced mitochondrial damage to innate immune sensing in copper-based nanotherapy (45, 46, 60). Reactive oxygen species may further contribute to inflammatory signaling and remodeling of the tumor immune microenvironment (45, 46).

The translational development of copper-based nanomedicine has rapidly advanced. Deng et al. reviewed nanoparticle strategies for inducing cuproptosis, highlighting challenges such as tumor-selective copper delivery and controlled intracellular accumulation, and proposing nanotechnological solutions including enhanced permeability and retention (EPR) effect, surface functionalization, and stimulus-responsive release systems (45). Zhang et al. further extended this framework by integrating copper-based cytotoxicity with metabolic reprogramming strategies for cancer immunotherapy (46).

On the design side, Meng et al. developed SCTDM, a biomimetic hybrid macrophage–tumor membrane platform co-loaded with the copper ionophore disulfiram and the lactate modulator syrosingopine, enabling synergistic induction of cuproptosis, photothermal therapy, and metabolic interference (47). Similarly, Cai et al. engineered a pH-responsive “cuproptosis switch” based on a bilayer copper nanocapsule containing Cu_2_O and Cu_2-x_Se shells, with the STING agonist MSA-2 encapsulated within; Cu^+^ release follows a switch-like profile across a transition pH of 6.8. Ultrasound irradiation together with MSA-2 release sensitized cells to cuproptosis by upregulating the copper influx transporter SLC31A1, downregulating the efflux transporter ATP7A, depleting glutathione, and increasing mitochondrial membrane permeability. Tumor-specific release of Cu^+^ and MSA-2, combined with ultrasound-induced ROS amplification, induced cuproptosis, immunogenic cell death, and cGAS–STING activation, achieving complete elimination of primary and distant tumors in mice at low doses (126). In this formulation, however, cGAS–STING activation is attributable at least in part to the co-delivered STING agonist MSA-2 and should not be interpreted as an intrinsic property of the copper nanocapsule.

Collectively, copper-based nanoparticles and nanoplatforms represent multifunctional therapeutic platforms that combine direct cytotoxicity via copper-dependent mitochondrial and redox mechanisms with innate immune activation through immunogenic cell death and inflammatory signaling amplification, positioning them as promising candidates for integrated cancer immunotherapy. The relative contribution of copper itself, nanoparticle architecture, and co-delivered immunostimulatory agents therefore needs to be considered when interpreting their innate immune effects.

### 4.6 Phytochemical nanoformulations

Encapsulating phytochemicals in nanocarriers overcomes several long-standing limitations of these compounds, including poor oral bioavailability, extensive first-pass metabolism, low aqueous solubility, and chemical instability. He et al. reviewed phytotherapeutic agents that modulate tumor-associated macrophages in colon carcinoma, identifying several distinct mechanistic axes of action (113).

A major regulatory node involves the NF-κB/STAT3 signaling axis, through which compounds such as curcumin and cucurbitacin B suppress M2-like macrophage polarization. This inhibition results in reduced VEGF (vascular endothelial growth factor)/HIF-1α (hypoxia-inducible factor-1α)-mediated angiogenesis and downregulation of immune checkpoint molecules such as PD-L1. Notably, curcumin-loaded nanocarriers, including liposomes, PLGA nanoparticles, and polymeric micelles, enhance these effects by improving stability and intracellular delivery, thereby potentiating anti-inflammatory and anti-tumoral signaling suppression (113, 127).

Additional phytochemicals act through complementary and partly independent immunomodulatory pathways. Sulforaphane and β-glucans modulate tumor-associated macrophage function through interactions with the gut microbiota and systemic immune signaling, linking the gut-immune axis to tumor inflammation. Astragaloside IV enhances innate antitumor immunity by promoting macrophage activation, whereas cannabidiol inhibits alternative macrophage activation and induces metabolic reprogramming toward a more glycolytic, antitumor phenotype, while hydroxygenkwanin promotes M1-like and suppresses M2-like macrophage polarization. In contrast, fucoidan attenuates pro-tumoral inflammation by inhibiting TLR4 signaling in macrophages, while dihydroisotanshinone I reduces tumor progression by suppressing CCL2-mediated macrophage recruitment (113, 128, 129).

Plant-derived vesicle-like nanoparticles (PVLNs) represent a parallel and increasingly important concept in phytonanomedicine. These naturally occurring nanostructures can be considered intrinsic delivery systems of plant bioactive molecules, combining carrier and cargo within the same biological entity. Their integration with isolated phytochemicals supports a broader conceptual framework of “phytonanomedicine,” in which plant-derived nanocarriers and phytochemical payloads act synergistically to modulate innate immune responses within the tumor microenvironment (108, 113). For phytochemicals delivered in lipid carriers, however, complement activation appears to depend strongly on carrier properties, particularly vesicle size. In a study of targeted nanoliposomes bearing different ligands, including curcumin derivatives, all targeted and PEGylated liposomes increased iC3b relative to PBS, but targeted formulations generally produced iC3b levels comparable to those induced by PEG-100 liposomes, whereas larger PEG-200 and PEG-400 liposomes produced substantially greater complement activation; both SC5b-9 and iC3b correlated positively with liposome diameter (130). In this system, therefore, complement activation was more strongly associated with vesicle size than with the presence of the phytochemical, although lipid composition and surface architecture may also contribute. This distinction between carrier- and cargo-driven activation applies across the classes considered here and is developed further below.

A direct quantitative comparison across these classes is not currently possible, for both biological and methodological reasons. PEG illustrates the difficulty: in lipid nanoparticles the PEG-lipid contributes to anti-PEG antibody responses, accelerated clearance and CARPA; in subcutaneous PLGA nanoparticles PEGylation instead reduces complement activation and neutrophil recruitment; and on gold surfaces it can abolish complement activation altogether. The immunological effect of PEGylation therefore depends on platform, surface architecture and route, and no uniform class-level interpretation is possible.

Between-donor variability exacerbates this problem. In a recent cohort, C3 deposition varied 26- to 29-fold between lowest- and highest-responding donors across two platforms, and deposition on the two correlated only weakly within the same donor (r = 0.31), indicating that complement activation is determined by the donor-platform combination rather than by either alone, so that donor ranking on one platform does not predict ranking on another; inflammatory status, steroid use, age, sex and C-reactive protein did not account for it. Pre-existing anti-PEG IgM was the strongest single predictor for PEGylated liposomal doxorubicin, whereas antidextran antibodies had no predictive value for iron oxide nanoworms; nanoparticle-bound immunoglobulins predicted best for both (131). Assays are themselves variable, with published and commercial anti-PEG ELISAs differing substantially in reagents and protocols (132), against a wider bio-nano literature heterogeneous enough to have prompted proposals for minimum reporting standards (133).

Cytokine responses, receptor engagement and complement activation are therefore comparable across classes at an ordinal level only, as summarized in **Table 5**, and the categories reported there are evidence-weighted profiles rather than intrinsic properties. Establishing relative immunological risk will require harmonized assays and recipient- and platform-specific stratification rather than class-level averages. Proposed SC5b-9 thresholds for estimating hypersensitivity risk remain dependent on assay, formulation and context (50, 131).

## 5 Nanoparticles and the tumor microenvironment: from immune remodeling to therapeutic perspectives

### 5.1 The immunosuppressive tumor microenvironment

The tumor microenvironment (TME) is a complex ecosystem whose immunosuppression is built from several overlapping layers, and it is the combined action, rather than any single mechanism, that blunts the response to immunotherapy. Much of the burden falls on the macrophage population, since M2-like tumor-associated macrophages (TAMs) dominate the infiltrate and release IL-10, TGF-β, and VEGF, sustaining both immune suppression and angiogenesis (23, 24, 134, 135). The T-cell compartment is compromised in parallel: persistent antigen exposure and inhibitory signaling drive the expression of PD-1, LAG-3, TIM-3, and TIGIT, while metabolic regulators such as adenosine signaling pathways contribute to adenosine-mediated immunosuppression, collectively locking effector T cells into a dysfunctional, exhausted state (25, 135). Metabolic reprogramming further reinforces this suppression. Hypoxia stabilizes HIF-1α and promotes a glycolytic shift, and the resulting accumulation of lactate acidifies the extracellular space to pH 6.5-6.8, a range that directly impairs effector immune cell function (134, 136). On top of these biochemical barriers sits a physical one: a dense, hyaluronic-acid-rich extracellular matrix that limits both drug penetration and immune cell infiltration, while a final checkpoint operates at the level of cell-cell recognition, as “don’t eat me” signals transmitted through the CD47/SIRPα axis restrain macrophage phagocytosis (23, 24). Because these suppressive mechanisms are highly redundant, inhibition of any single pathway is often insufficient, which is precisely why multimodal strategies, including nanoparticle-based approaches, are increasingly explored for TME reprogramming (135).

### 5.2 Nanoparticle strategies for TME remodeling

The defining advantage of nanoparticles in this setting is multivalency. Unlike a free small molecule, which typically acts on a single target, one nanoparticle platform can be engineered to engage several immunosuppressive axes at once, either by carrying multiple payloads or by combining an intrinsic material property with a loaded drug (17, 27, 30, 31, 60). The strategies developed along these lines tend to converge on a common goal: shifting the tumor from an immunologically silent state to one in which an antitumor response can be mounted.

The most extensively investigated of these approaches works through the cGAS–STING pathway. When STING agonists are encapsulated in lipid nanoparticles or polymersomes, or when Mn²^+^ ions are released from metallic nanoparticles (rather than Cu²^+^ acting as a direct STING activator), the resulting rise in type I interferon and dendritic cell maturation promotes cytotoxic T-cell infiltration into tumors that were previously immune-excluded: a process often described as the transition from a “cold” to a “hot” tumor phenotype. Ying et al. and Jiang and Lin have mapped these delivery routes in detail, and Shaik et al. extended the picture to the preclinical and clinical progress of STING agonists in nanoparticle form (17, 27, 30).

Closely related is the effort to reprogram the macrophage compartment, where nanoparticles carrying TLR agonists, STAT3/NF-κB inhibitors, or phytochemicals such as curcumin can push tumor-associated macrophages away from their immunosuppressive M2-like phenotype and toward a tumoricidal M1-like state, tilting the local balance from tumor support to tumor control (23, 24, 32, 113, 137).

A distinct line of work exploits the ability of nanoparticles to kill tumor cells in a way that is immunologically visible. Photothermal therapy with gold nanorods, copper systems that induce cuproptosis primarily through mitochondrial proteotoxic stress and ROS generation (rather than direct STING activation), chemotherapy delivered through docetaxel-loaded polymeric platforms, and photodynamic therapy all provoke immunogenic cell death, releasing danger-associated molecular patterns that restart the antitumor immune cycle (44, 45, 116, 138). Where the obstacle is physical rather than biochemical, nanoparticles can instead be used to dismantle the stroma directly: nanocarriers co-delivering matrix-degrading enzymes such as hyaluronidase can loosen the dense, hyaluronic-acid-rich stromal extracellular matrix, improving both drug penetration and immune-cell access to the tumor core. In one such approach, hyaluronidase packaged within metal–polyphenol nanoparticles and released *in situ* by a clinically used iron chelator degraded the tumoral matrix and markedly increased intratumoral infiltration of cytotoxic T lymphocytes following photothermal and photodynamic therapy (139). **Table 6** summarizes these strategies together with the nanoparticle classes involved and their principal preclinical outcomes.

**Table 6:** Nanoparticle strategies for tumor microenvironment remodeling.

Strategy	NP class	Immunological mechanism	Key preclinical result	Ref.
cGAS–STING activation	LNP, AuNR/MnO_2_, Cu NP	IFN-I, DC maturation, CD8^+^Tcell priming	Cold→hot conversion; enhanced CD8^+^ infiltration	(27, 30)
TAM repolarization	Polymers, phytochemical NPs, exosomes	M2→M1 via NF-κB/STAT3	TME immunosuppression reduction	(23, 24, 110, 113)
ICD (photothermal)	AuNR, AuNS	DAMP release (CRT, HMGB1, ATP), DC activation	High tumor inhibition and abscopal responses	(117, 120)
ICD (cuproptosis)	Cu-based NPs	Mitochondrial stress-ROS, DAMPs, STING activation	Primary + metastatic tumor regression in preclinical models	(44–46)
ECM modulation	Metal–polyphenol NPs	Hyaluronidase; immune infiltration	Improved immune and drug penetration in solid tumors	(139)
Phagocytosis reactivation	exosomes	CD47/SIRPα axis blockade → macrophage activation	Synergism with immunotherapy; enhanced phagocytosis	(109, 110)
Vascular normalization	LNP, polymers	Anti-VEGF; hypoxia reduction	Increased immune infiltration	(31)
mRNA nanovaccines	LNP	DC activation; antigen expression; CD8^+^ T priming	Neoantigen-specific adaptive responses	(14, 16, 100)

Entries list strategies with preclinical support; clinical status is indicated where applicable.

### 5.3 Future perspectives and emerging directions

Looking beyond the strategies already validated in preclinical models, several directions are beginning to take shape, and they differ markedly in how close they are to the clinic. The most computationally driven of them is the use of artificial intelligence in formulation design. Machine learning and deep learning models are now being trained to predict how a given nanoparticle will behave immunologically, to fine-tune formulation parameters, and to support patient-specific design, and this approach is gaining ground quickly as the underlying datasets grow (52, 140, 141). A parallel trend is the move toward multimodal platforms that integrate several treatment modalities into a single construct: photothermal therapy, immunotherapy, chemotherapy, and epigenetic modulation combined in one system. The GS@AM@M platform for photothermal epigenetic regulation and the SCTDM system for combined induction and lactate metabolism targeting illustrate this “all-in-one” nanoplatform logic (47, 121). In parallel, implantable polymer-based systems such as μMESH (142) extend this concept to spatially controlled, local chemo-immunotherapy, representing a device-level rather than nanoparticle-level integration strategy.

The scale this approach has reached is worth stating concretely. Starting from a combinatorial library of 584 ionizable lipids screened for transfection potency, a gradient-boosting model trained on those data was used to search a virtual library of 40,000 candidates; the sixteen top-ranked structures were synthesized, and one of them outperformed both benchmark lipids used in approved products for intramuscular delivery in the experimental model (143). The design space of programmable lipid nanoparticles has likewise been mapped into discrete levers with defined trade-offs, including apparent pKa, linker degradability, helper-lipid and sterol identity, PEG-anchor length and the addition of a fifth selective-organ-targeting lipid, with immune interactions listed among the properties these levers are meant to control (144). What such pipelines optimize, however, is almost always a single objective. In the study above the training label was transfection potency reduced to a binary threshold, and the preferential transfection of hepatic and splenic immune cells by the selected lipid emerged from characterization after selection rather than from the selection criteria themselves. A property of that kind can be an opportunity or a liability depending on the indication, and an optimization that does not represent it cannot distinguish between the two.

Integrating mechanistic immunology into this workflow means moving immunological endpoints from downstream characterization into the objective function itself, a direction already articulated in proposals for generative models that would maximize cell-specific delivery while minimizing immunotoxicity, within closed-loop cycles coupling automated synthesis, characterization, screening and machine-learning optimization (145).

The components are largely available. Physiologically based pharmacokinetic modeling already bridges immune variables and nanoparticle disposition: a minimal model incorporating anti-PEG antibody kinetics reproduced the clinical pharmacokinetics of pegloticase and predicted that prolonged circulation is compromised only above approximately 500 ng/mL of circulating antibody (146): the threshold exceeded by only a small minority of the population, as noted in Section 3.5. Parameter-space sampling constrained by PET/CT biodistribution data then localized the effect, identifying hepatic retention as its primary and splenic retention as its secondary determinant (147, 148). Multiscale molecular dynamics and generative models are being applied to the same design space (149), and the endpoints that would populate an objective function are those discussed throughout this review: receptor-level activation by ionizable lipids, complement deposition, markers of innate memory.

The obstacle is empirical. Transfection data are generated systematically within single campaigns, whereas immunological data must be assembled retrospectively: a gradient-boosting model predicting vaccine-induced IgG titers was trained on 325 LNP formulations with associated antibody titers, reaching a coefficient of determination above 0.87 and recovering known critical substructures of ionizable lipids (150). Such models inherit the heterogeneity of their sources, and that heterogeneity has been measured directly: in a multicenter study, distributing uniform protein corona digests and imposing standardized preparation, comparable instrumentation and a unified database search raised the overlap in protein identifications between facilities from 11% to 40% (151). Design criteria so derived risk resting on correlation rather than causation (152), and no published application of machine learning to the direct optimization of lipid nanoparticle formulations for biological performance in humans has been identified, a gap attributed to data scarcity and limited transparency of complete datasets (153). Prospective generation of harmonized immunological datasets is therefore a prerequisite rather than a parallel concern, and integrated pipelines combining automated synthesis, high-throughput characterization and multiplexed in vivo barcoding provide the infrastructure through which they could be produced (145). Mechanistic models would then constrain and interpret the learned relationships, distinguishing causal design variables from formulation-specific correlations.

A further target that illustrates the same logic is TRIM29, which sits at the intersection of oncogenic signaling and innate immune regulation. It is overexpressed in lung, bladder and pancreatic cancers, where it potentiates oncogenic pathways and promotes metastatic progression and therapeutic resistance, but its function is context-dependent and tumor-suppressive roles have been documented in breast and esophageal cancers (154). As a regulator of innate signaling it acts through several distinct mechanisms: in alveolar macrophages it restrains type I interferon and proinflammatory cytokine production by K48-linked ubiquitination and degradation of NEMO (155); in airway epithelial cells and myeloid dendritic cells it is induced by cytosolic double-stranded DNA and targets STING for ubiquitination at Lys370, blunting the cGAS-STING axis (156); in cardiomyocytes it functions instead as a SUMO E3 ligase, recruiting Ubc9 to SUMOylate and stabilize PERK, sustaining endoplasmic reticulum stress and the accumulation of immunosuppressive monocytic myeloid-derived suppressor cells (157); and in intestinal epithelial cells it degrades NLRP6 and NLRP9b, suppressing type III interferon and inflammasome-derived IL-18 (158).

Because these pathways all influence antitumor immunity, TRIM29 is a plausible immunomodulatory target, but the evidence derives largely from antiviral, inflammatory or non-tumor models and the direction of intervention cannot be assumed. Blocking the STING-TRIM29 interaction raises cellular STING levels, and the small-molecule inhibitor SB24011 potentiated both a STING agonist and an anti-PD-1 antibody in vivo (159). In gastric cancer the relationship is inverted: TRIM29 is expressed at lower levels in tumor than in paired normal tissue, in association with CpG-island hypermethylation, and where present it ubiquitinates IGF2BP1 at Lys440 and Lys450, destabilizing PD-L1 mRNA, so that TRIM29 levels correlate with CD8+ T-cell infiltration and overall survival. Yet TRIM29 depletion synergized with PD-1 blockade rather than proving detrimental, and silencing it did not alter tumor growth in immunodeficient mice lacking transferred lymphocytes, indicating a predominantly immune-mediated role; the authors proposed the TRIM29/IGF2BP1/PD-L1 axis as a biomarker and therapeutic target (160). The direction of intervention therefore depends on tumor type, on which substrate dominates, and on the therapeutic context.

To our knowledge no nanomedicine approach targeting this axis has been reported, although two delivery problems would lend themselves to one. TRIM29 is a RING-less TRIM protein and a protein-protein interaction regulator, so direct pharmacological targeting is challenging and nucleic-acid-based modulation is the tractable route where inhibition is desirable. The small-molecule route has the converse difficulty: SB24011 is efficacious but was administered intratumorally throughout, and a plasma half-life of approximately 0.26 h with low exposure and low microsomal stability led the authors to exclude a major contribution of circulating compound to the distal-tumor effect (159). Extending half-life and directing biodistribution are problems nanocarriers are designed to address, and cell-selective delivery would mitigate the principal objection to systemic modulation of a protein with opposite functions in different tissues. This remains a hypothesis rather than an established strategy.

The direction with the firmest clinical footing is personalized nanovaccines, which builds directly on the validated mRNA–LNP platform: mRNA encoding a patient’s own tumor neoantigens, paired with an optimized adjuvant lipid nanoparticle, is now among the most actively pursued strategies in immuno-oncology (14–16, 71, 101). Less developed, but conceptually intriguing, is the interface between nanoparticles and the gut microbiome, where the interplay among nanomaterials, microbial communities, and innate immunity is emerging as a regulatory axis of particular relevance to nanoformulated phytochemicals and dietary immunomodulators (113). The last direction brings the review back to a theme that has surfaced repeatedly: the deliberate induction of trained immunity in tumor-associated macrophages, aimed at converting a transient immunosuppressive state into durable antitumor responsiveness. This remains one of the least explored frontiers in cancer nanomedicine, and arguably one of the most promising (20–22). Collectively, these directions highlight the convergence of computational design, systems immunology, and multifunctional nanoplatform engineering in shaping next-generation cancer immunotherapy.

## 6 Challenges for clinical translation

Translating immunomodulatory nanomedicines into clinical practice raises difficulties that go beyond those facing conventional nanomedicines (5, 52, 161). Several of these deserve particular attention, and they are best understood not as isolated technical hurdles but as an interconnected set of problems that begin at the bench and extend to the regulatory approval process.

The first concerns the way immune characterization is performed. The field still lacks a harmonized panel of assays for assessing nanoparticle immunogenicity across laboratories, materials, and platform classes, and this absence has practical consequences. The line separating beneficial from adverse immune activation is highly context-dependent, and drawing it reliably requires functional assays that current regulatory frameworks have not yet codified (5, 7, 8). A related difficulty surrounds the biomolecular corona. Most studies characterize it in static plasma or serum, yet the corona that forms in vivo is dynamic and context-dependent: it shifts with blood flow, varies between tissues, and reflects each patient’s pathological state. Until corona analysis is standardized under physiologically realistic conditions, predictions based on static measurements will remain provisional (56, 57).

Time introduces a third problem. Nanoparticle-induced trained immunity can reshape immune responses over weeks to months, but the experimental window rarely matches that timescale: most preclinical studies provide follow-up of only four to eight weeks, leaving the longer-term consequences largely undocumented (20, 21).

Manufacturing poses a fourth, more tangible barrier. Producing complex nanomedicines such as multi-compartment systems, biomimetic constructs, and biologic-loaded particles under good manufacturing practice (GMP) conditions remains a serious bottleneck, and here the contrast with lipid nanoparticles is instructive, since LNP production already benefits from scalable modular manufacturing platforms (including microfluidic systems) validated in mRNA vaccine manufacturing (5, 52, 161).

A fifth difficulty concerns the mitigation of PEG immunogenicity itself. The strategies that have been proposed are summarized in **Table 7**, ordered by the strength of the evidence supporting them. Although clinical evidence indicates that anti-PEG immunity can affect the pharmacokinetics of PEGylated therapeutics in selected settings (167), clinical evidence that any specific mitigation strategy prevents or reduces ABC in patients remains limited. The best-documented approaches modify the dosing regimen (92) or the architecture of the polymer rather than replacing it (162); replacement and the more recent proposals are supported by preclinical data or by no primary comparative data at all.

**Table 7:** Proposed strategies to mitigate PEG immunogenicity, with the evidence supporting each and its principal limitation.

Strategy	Evidence	Limitation	Ref.
Dosing interval	ABC strongest at 3 days for PEGylated cerasomes; 5-day peak for PEGylated liposome controls; absent at 14 days	Rat data; optimal interval not validated clinically	(92)
Surface charge	Negatively charged PEGylated cerasomes showed reduced ABC compared with neutral cerasomes (ABC index 0.129 vs 0.766)	Rat data; mechanism underlying charge dependence remains incompletely resolved	(92)
PEG architecture	Antibodies to the terminal methoxy group do not accelerate clearance of single-chain or branched conjugates; effect grows with number of chains	Defined with monoclonal antibodies, not patient sera	(162)
Free PEG as decoy	Pre-infusion restored exposure of a PEGylated enzyme to ~80% of naive values in sensitized mice	Murine model	(163)
Polymer replacement (polysarcosine)	Full substitution of PEG lipid maintained or increased mRNA delivery with comparable safety	Anti-polymer responses not measured directly	(164)
Cleavable PEG linkers	Proposed	Comparative data on reduced ABC remain limited	(165)
Zwitterionic coatings	Proposed	Comparative data on reduced ABC remain limited	(165)
Risk-assessment frameworks	Published for nucleic acid therapeutics	Explicitly exclude applications where an adaptive response is intended	(166)

No strategy has yet been evaluated against a clinical outcome.

The final difficulty emerges only at the clinical endpoint. For immuno-oncological nanomedicines that pair direct cytotoxicity with immune modulation, conventional radiological criteria such as Response Evaluation Criteria in Solid Tumors (RECIST) may miss part of the therapeutic effect; phenomena like delayed responses and pseudoprogression underscore the need for validated immunological surrogate endpoints (52, 161).

Beyond these scientific and technical barriers, translational progress is also limited by regulatory and organizational fragmentation. Writing on behalf of the European Technology Platform on Nanomedicine, Germain et al. noted that although more than fifty nanomedicine formulations are already marketed and over four hundred are undergoing clinical evaluation, fragmented regulatory and standardization frameworks continue to hinder clinical translation. To address this challenge, they advocated the creation of a common European knowledge hub linking academia, industry, healthcare providers, and policy makers to improve coordination and accelerate translation (5).

## 7 Conclusions

This review has traced the interactions between nanoparticles and the innate immune system across six major classes (liposomes and LNPs, exosomes and PVLNs, polymeric nanocapsules, gold nanoparticles and nanorods, copper-based systems, and phytochemical nanoformulations), highlighting both the mechanistic principles they share and the features that distinguish them. Three conclusions emerge from this comparison.

The first is that the interaction between nanoparticles and the innate immune system is not simply an adverse effect to be minimized but a fundamental biological phenomenon that governs the safety, efficacy, and therapeutic potential of every nanomedicine. The old dichotomy between “desired” immunogenicity, as in vaccinology, and “undesired” immunogenicity, as in drug delivery, is steadily giving way to a single framework in which immunological behavior is treated as a core design parameter, on the same footing as size, charge, and release kinetics. The second is that bringing nanotechnology and innate immunology together has made possible therapeutic strategies that were barely conceivable a decade ago: TAM repolarization, cGAS–STING activation through metallic nanoparticles, immunogenic cuproptosis, photothermal and photodynamic immunotherapy, and the modulation of trained immunity. In all of these strategies the nanoparticle acts not only as a delivery vehicle but as an immunological agent in its own right. The third is that the field still lacks a unifying conceptual and methodological framework because knowledge remains fragmented across nanotechnology, immunology, oncology, pharmacology, and regulatory science. The lack of standardized immunological characterization protocols and of validated clinical endpoints for immunomodulatory nanomedicines remains among the major barriers to clinical translation. Three requirements follow from the evidence reviewed here. Attributing an innate response to a nanomaterial requires controls that distinguish the carrier from its cargo and from contaminants, and this distinction is still applied inconsistently. Inter-individual variability is not residual noise but a determinant of outcome, as shown by the range of complement responses between donors, the donor-dependence of innate memory profiles, and the confinement of antibody-mediated clearance to a minority with high titers; recipient stratification is therefore likely to matter as much as platform selection, although it must be established for each platform rather than as a general risk profile. And the integration of mechanistic immunology with multiscale modeling and data-driven design, now within reach, depends on immunological datasets generated prospectively to a common standard rather than aggregated retrospectively.

The comparison across classes also makes clear where the most productive work now lies, pointing to six areas in particular. The first is immunogenicity and safety: the systematic profiling of immunogenicity across classes, the development of standardized immunoassays, the characterization of the biomolecular corona under physiologically relevant conditions, and the long-term evaluation of immune-related nanotoxicology. The second is mechanistic: dissecting how different nanoparticle classes activate PRR pathways such as TLR, cGAS–STING, and the NLR/inflammasome axis, mapping the epigenetic and metabolic reprogramming behind trained immunity, and elucidating the innate–adaptive crosstalk that nanomaterials set in motion. The third concerns clinical translation in oncology, leveraging these mechanisms into TME remodeling, pairing immunogenic cell death with STING activation, reversing T-cell exhaustion, and combining chemotherapy, photothermal therapy, and immunomodulation.

The remaining three are equally important. One is the design of specialized nanocarriers in which immunological behavior is built in from the start (multi-compartment polymeric systems, biomimetic vesicles, LNPs engineered with adjuvant properties, and stimuli-responsive platforms for tumor-specific delivery). Another is phytonanomedicine, the systematic study of nanoformulated phytochemicals and PVLNs for immunomodulation, with particular attention to TAM reprogramming, microbiome–immune crosstalk, and the synergy between plant-derived carriers and bioactive compounds. The last concerns the regulatory and clinical implementation of nanomedicines, including the development of standardized immunological testing frameworks, clinically meaningful endpoints for immunomodulatory therapies, harmonized EMA/FDA regulatory pathways for drug–device hybrids, and the interdisciplinary consortia needed to advance them.

Immune-aware nanomedicine sits at an inflection point. Its conceptual foundations are solid, its preclinical evidence is extensive, and the clinical success of mRNA–LNP vaccines has already shown what nanoparticle-innate immunity interactions can achieve. What remains is the harder task of turning a fragmented body of knowledge into a coherent, predictive and operational basis for the rational design of next-generation immunomodulatory nanomedicines. By integrating these perspectives, this review aims to contribute to that convergence and to support the interdisciplinary framework required for immunologically informed nanomedicine to realize its full clinical potential.
