# Supplementary material for: Nanomedicine and the innate immune system: immunogenicity, nanoparticle-immune cell interactions and implications for cancer immunotherapy
Source: Front Immunol. 2026 Oct 20;17:1946595. doi: 10.3389/fimmu.2026.1946595 (PMC13729012; doi:10.3389/fimmu.2026.1946595)
Supplement: Supplementary file 1 [file DataSheet1.pdf]

Supplementary Material

Nanomedicine and the Innate Immune System: Immunogenicity, Nanoparticle-Immune Cell Interactions and Implications for Cancer Immunotherapy

Giuseppina Bozzuto[1†], Sofia Migani [1,2], Marisa Colone[1], Annarica Calcabrini[1], Annarita Stringaro[1†*] and Maria Luisa Dupuis[1*]

[1] National Center for Drug Research and Evaluation, Italian National Institute of Health, Viale Regina Elena, 299, 00161 Rome (Italy)

[2] School of Science and Technology, Chemistry Division, University of Camerino, via Madonna delle Carceri (ChIP), 62032 Camerino (MC) (Italy)

Supplementary Methods. Literature search strategy

PubMed searches were conducted from January 2003 to September 2026. Searches combined terms describing each nanoparticle class (liposome, lipid nanoparticle, exosome, plant-derived vesicle, polymeric nanoparticle, gold nanoparticle, gold nanorod, copper nanoparticle, phytochemical nanoformulation) with terms describing innate immune mechanisms (innate immunity, pattern recognition receptor, Toll-like receptor, cGAS–STING, inflammasome, complement activation, macrophage polarization, dendritic cell, natural killer cell, neutrophil extracellular traps, mast cell, trained immunity, immunogenic cell death) and, where relevant, with terms relating to the tumor microenvironment and cancer immunotherapy. Reference lists of relevant retrieved articles were also screened to identify additional primary studies and relevant reviews. Retrieved records were screened for relevance based on title and abstract, followed, where appropriate, by assessment of the full text against the eligibility criteria.

Records were considered eligible when they reported original experimental data or relevant review-level synthesis concerning the interaction between engineered nanomaterials and innate immune effectors, were published in English, and provided sufficient methodological information to identify the experimental system and nanomaterial formulation. Records were excluded when the nanomaterial was insufficiently characterized, when immunological readouts could not be attributed to a defined formulation, or when nanomaterial toxicity was investigated without a defined immune component. The same eligibility framework was applied across nanoparticle classes and innate immune pathways, while the search terms were adapted to each nanoparticle class and immune mechanism. Primary studies were prioritized for mechanistic claims, whereas review articles were used to synthesize broader bodies of evidence that could not reasonably be represented by individual primary studies.

This is a narrative and comparative review rather than a systematic review. Its aim is to identify mechanistic principles that recur across nanoparticle classes and to examine the conditions under which these principles may or may not apply, rather than to answer a single pre-specified question through exhaustive literature retrieval. No formal risk-of-bias assessment, quantitative

evidence synthesis, or meta-analysis was undertaken. Accordingly, a PRISMA flow diagram was not included, as the present review was not designed as a systematic review. Where the evidence base for a given statement is limited, the experimental model, nanoparticle formulation, and level of evidence are specified in the main text and, for the cross-platform comparison, in Table 5.
